# Outer membrane vesicles derived from probiotic *Escherichia coli* Nissle 1917 promote metabolic remodeling and M1 polarization of RAW264.7 macrophages

**DOI:** 10.3389/fimmu.2025.1501174

**Published:** 2025-05-29

**Authors:** DongXue Ma, YuXin Zhang, Jia Zhang, Jun Shi, ShanHu Gao, Fei Long, Xin Wang, XingYu Pu, Jiayao Sun, Shuang Liang, Richard D. Cannon, Silas Villas-Boas, Ting-Li Han

**Affiliations:** ^1^ Department of Obstetrics and Gynecology, The Second Affiliated Hospital of Chongqing Medical University, Chongqing, China; ^2^ State Key Laboratory of Ultrasound in Medicine and Engineering, College of Biomedical Engineering, Chongqing Medical University, Chongqing, China; ^3^ Department of Oral Sciences, Sir John Walsh Research Institute, Faculty of Dentistry, University of Otago, Dunedin, New Zealand; ^4^ Environmental Research and Innovation Department, Luxembourg Institute of Science and Technology, Esch-sur-Alzette, Luxembourg

**Keywords:** *Escherichia coli* Nissle 1917, outer membrane vesicles (OMVs), macrophage polarization, metabolomics, fluxomics

## Abstract

**Introduction:**

*Escherichia coli* Nissle 1917 (EcN) is one of the most extensively studied nonpathogenic Gram-negative probiotic strains worldwide. Recent research has highlighted the ability of EcN outer membrane vesicles (OMVs) to enhance the phagocytosis and proliferation of RAW264.7 macrophages. However, the impact of EcN-OMVs on M1/M2 polarization and metabolic modulation remains unknown.

**Methods:**

In this study, we evaluated the metabolic effects of EcN-OMVs on RAW264.7 macrophage polarization using metabolomic, transcriptomic, and fluxomic approaches.

**Reuslts:**

We found that the RAW264.7 macrophages phagocytosed EcN-OMVs, triggering upregulation of the HIF-1, mTORC1, and NF-κB signaling pathways. This metabolic reprogramming enhanced glycolysis, suppressed the TCA cycle, elevated intracellular reactive oxygen species (ROS), TNF-α, IL-6, IL-1β, ATP, and nitric oxide (NO) production, and promoted macrophage proliferation, migration, invasion, and M1-type polarization.

**Discussion:**

In summary, this research establishes a theoretical foundation for utilizing probiotic OMVs in immunomodulatory therapeutic applications.

## Graphical Abstract

The effect of *Escherichia coli* Nissle 1917-derived outer membrane vesicles (OMVs) on RAW264.7 macrophages. *Escherichia coli* Nissle 1917 OMVs are internalized by RAW264.7 macrophages, triggering M1 polarization through key metabolic pathways, including glycolysis and the TCA cycle. Metabolic intermediates such as branched-chain amino acids (BCAAs), stearic acid, and succinic acid activate mTORC1 and HIF-1α signaling pathways, leading to increased production of pro-inflammatory cytokines (TNF-α, IL-6, and IL-1β), reactive oxygen species (ROS), nitric oxide (NO), and lactate. These metabolic and signaling changes enhance macrophage functions such as phagocytosis, proliferation, migration, and invasion, highlighting the immunomodulatory potential of OMVs.

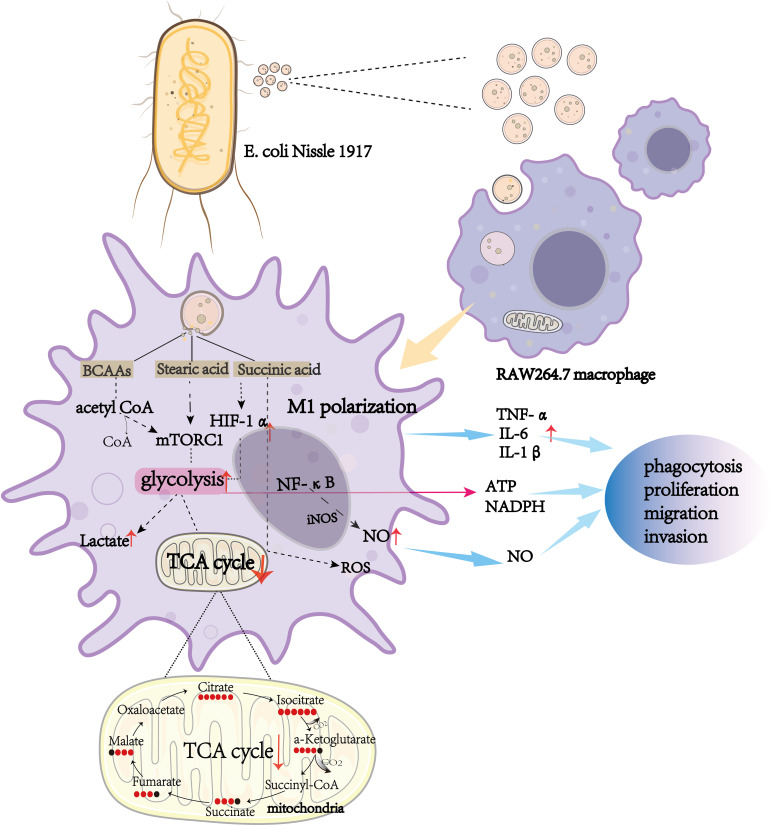

## 1 Introduction

*Escherichia coli* Nissle 1917 (EcN) is a nonpathogenic Gram-negative strain and one of the most extensively studied probiotic bacteria (1). One characteristic of Gram-negative bacteria is the production of outer membrane vesicles (OMVs) (2, 3), which are spherical structures derived from the outer membrane that are gaining recognition for their important role in mediating communication between bacteria and immune cells (4–6). OMVs can deliver effector molecules in a stable and efficient manner directly to immune cells and affect their biological functions (2). Some studies have reported that EcN-derived OMVs (ECN-OMVs) have immunomodulatory effects on human peripheral blood mononuclear cells and induce cytokine secretion (3). Moreover, ECN-OMVs have been shown to mediate anti-inflammatory effects and in inflammatory bowel disease cause infiltration of macrophages which relieves ulcerative colitis (4). As EcN-OMVs can have significant effects on macrophages it is important to understand the mechanism of the interaction between EcN-OMVs and macrophages as this might indicate how EcN-OMVs can be used clinically to maintain immune homeostasis.

Macrophages are considered a crucial component of innate immunity and are vital in the inflammatory response (6). Macrophages display two distinct phenotypes; namely M1 and M2 macrophages (7, 8). M1-polarized macrophages are immune-effector cells that exhibit antimicrobial and anti-tumor properties by producing pro-inflammatory and immune-stimulating cytokines such as interleukin-6 (IL-6), interleukin-15 (IL-15) (9), tumor necrosis factor-α (TNF-α), and cytokine-inducible nitric oxide synthase (iNOS) (10–12). Conversely, M2 macrophages release anti-inflammatory cytokines, including interleukin-4 (IL-4), interleukin-10 (IL-10), and transforming growth factor β (TGF-β), which suppress immune responses and help to regulate inflammation (10–12). Macrophage polarization is often accompanied by a shift from glycolysis to oxidative phosphorylation (13–15). Indeed, M1 macrophages rely on glycolysis along with the pentose phosphate pathway for their metabolic activity (16, 17) and to meet their ATP requirements, whereas flux through the Krebs cycle is reduced at two steps (conversion of isocitric acid to α-ketoglutaric acid, and succinic acid oxidation) (17, 18), and there is downregulation of oxidative phosphorylation (OXPHOS) and fatty acid oxidation (FAO) (17, 19, 20). In contrast, in M2 macrophages there is normal flux through the Krebs cycle, and their metabolic activities are characterized by enhanced FAO and OXPHOS (17). In both M1 and M2 macrophages, reprogramming of amino acid metabolism and ferroptosis are also observed (21). Emerging evidence suggests that aquaporin-9 (AQP) plays an important role in the priming of myeloid cells (5) and influences the phagocytic capacity and polarization of macrophages (22, 23). Moreover, intercellular adhesion molecule-1 (ICAM-1) has been identified as a critical mediator of phagocytosis in human leukocytes, including neutrophils (25) and macrophages (24). Importantly, extracellular vesicles have been reported to regulate ICAM-1 expression in leukocytes (25) and modulate the immune microenvironment (26). These findings suggest that OMVs may promote macrophage phagocytosis via ICAM-1, warranting further investigation. Recently, a study has shown that EcN-OMVs enhance RAW264.7 macrophage phagocytosis and proliferation (27), but their effects on M1/M2 polarization and metabolic activities remain unknown.

In the present study, we employed metabolomic and transcriptomic approaches to elucidate the mechanism by which EcN-OMVs affect macrophage polarization. We used isotope tracer experiments and orthogonal functional analysis to clarify the underlying metabolic flows in the central carbon pathways and energy metabolism of macrophages in response to EcN-OMVs.

## 2 Materials and methods

### 2.1 Cell culture

RAW264.7 murine macrophages were purchased from the Cell Bank of the Chinese Academy of Sciences (Shanghai, China). Before use in experiments, the cell line underwent testing for mycoplasma contamination. RAW264.7 macrophages (5–10 cell passage number) were cultured in Dulbecco’s Modified Eagle Medium (DMEM) (HyClone, USA), supplemented with 10% fetal bovine serum (FBS) (Gibco, AUS) and 1% penicillin–streptomycin (Sigma-Aldrich, St. Louis, MO, USA), at 37°C and 5% CO_2_. The culture medium was replaced every 24 h and the cells were passaged every 48 h.

### 2.2 Bacterial strains and isolation of OMVs

*Escherichia coli* Nissle 1917 (EcN) was obtained from Biobw (GmbH, Beijing, China), and cultured in Luria-Bertani broth at 37°C with continuous aeration through shaking at 180 rpm. To isolate OMVs, the bacteria were grown for 14 h to late log phase (OD_600_ of 0.9 to 1.0) (28). The culture was centrifuged at 5,000 g (Beckman Coulter, Brea, CA, USA) for 30 min at 4°C to remove the bacteria and then the supernatant was centrifuged at 10,000 g for 30 min at 4°C to remove remaining cell debris and macropolymers. The supernatant was filtered (0.45 μm pore size membrane; Millipore, MA, USA) and the sample was concentrated using an ultrafiltration device equipped with a 100 kDa Hollowfiber membrane (Amersham Biosciences). The concentrate was subsequently filtered through a 0.22 μm pore size membrane (Millipore) and was centrifuged at 160,000 g (Optima L-100XP ultracentrifuge, Beckman Coulter, USA) for 3 h at 4°C. The pellet was washed and resuspended in sterile phosphate buffered saline (PBS; pH 7.4) and centrifuged again at 160,000 g for 3 h at 4°C. The purified EcN-OMVs were uniformly dispersed in sterile PBS and stored at -80°C for future use. The protein content of isolated EcN-OMVs was measured using a BCA Protein Assay Kit (Beyotime, Shanghai, China).

### 2.3 EcN-OMV characterization

Nanoparticle Tracking Analysis (NTA) was employed to assess the concentration and size distribution of EcN-derived OMVs. EcN-derived OMVs were resuspended in 1 mL of phosphate-buffered saline (PBS) and applied to the NS300 nanoparticle analyzer (Malvern, Worchestershire, UK). The size of EcN-OMVs was calculated based on Brownian motion. OMVs were fixed with 2.5% glutaraldehyde and then spotted onto 300-mesh formvar/carbon-coated grids for TEM observation. EcN-derived OMVs were negatively stained with aqueous phosphotungstic acid for 1 min at room temperature and subsequently visualized using a transmission electron microscope (JEM-1400PLUS, JEOL, Japan).

### 2.4 Western blotting

EcN-OMVs or cells were lysed in 100 µL of RIPA buffer (Beyotime) containing 1% phenylmethylsulfonyl fluoride (PMSF) (Beyotime). After protein quantification using a BCA assay, samples (30 μg of total protein) were separated on 12% polyacrylamide gels and transferred to polyvinylidene fluoride (PVDF) membranes (Millipore). The membranes were blocked with 5% skimmed milk for 1 h at room temperature and incubated overnight at 4°C with the following primary antibodies: anti-iNOS (Affinity Biosciences, USA), anti-OmpA (Proteintech, USA), anti-OmpC (Proteintech, USA), anti-beta Actin (Affinity Biosciences, USA), anti-HIF-1α (Affinity Biosciences, USA), and anti-p65 (Affinity Biosciences, USA). After incubation with the corresponding horseradish peroxidase-conjugated secondary antibodies (Beyotime; diluted 1:5,000), the chemiluminescence signal was detected using an enhanced chemiluminescence detection reagent (Sigma-Aldrich, St. Louis, MO, USA), and images were acquired using a gel imaging system (Azure C400, Biosystems, USA).

### 2.5 Visualization of EcN-OMV uptake by RAW264.7 macrophages

To evaluate whether EcN-OMVs were taken up by macrophages, EcN-OMVs were labeled with the fluorescent dye PKH26 (Umibio, Shanghai, China) following the manufacturer’s instructions. In summary, the purified OMVs were resuspended in 200 μL PBS containing 100 μM PKH26 and incubated for 10 min at 37°C. RAW264.7 macrophages (1×10^5^ cells) were seeded onto a glass slide and then co-cultured with PKH26-labelled EcN-OMVs at 37°C and 5% CO_2_ for 24 h. After the incubation, the macrophages were washed three times with PBS and fixed with 4% paraformaldehyde for 30 min, and then permeabilized with 0.3% TritonX-100 (Sigma-Aldrich) for 20 min at 4°C. The cells were then blocked with PBS containing 5% bovine serum albumin for 2 h at room temperature. Cell membranes were immunoassayed with anti-F4/80 Ab (1:1000, Golbal, MO, USA), followed by Dylight Fluor-conjugated Goat Anti-Rat IgG (1:2000, EarthOx, LLC, San Francisco, CA, USA). The nucleus was labelled with 4, 6-diamidino-2-phenylindole (DAPI) (Beyotime) for 15 min and washed twice with PBS before confocal microscopy (TCS SP2, Leica) imaging.

### 2.6 Cell proliferation assay

RAW264.7 cells were seeded in 96-well microtiter plates (flat, tissue culture (TC)-treated, 2×10^4^ cells/well) and incubated with various concentrations of EcN-OMVs (0.1, 1 and 10 μg/mL) or vehicle control (PBS) at 37°C with 5% CO_2_ for 24 h. The cell viability was then measured using a CCK-8 cell viability assay kit following the manufacturer’s instructions (Dojindo, Shanghai, China). Each treatment group comprised six replicates, and three independent experiments were performed.

### 2.7 Phagocytosis assay

RAW264.7 cells (5 × 10^4^ cells per well) were cultured with EcN-OMVs (1 μg/mL) or PBS at 37°C with 5% CO_2_. After 24 h incubation, 100 μL 0.1% neutral red solution (Aladdin, Shanghai, China) was added to each well, and the cells were incubated for 30 min, after which the culture medium was removed and the cells were washed three times with PBS. The cells in each well were lysed with 100 μL ethanol:acetic acid (1:1) and the absorbance was measured at 450nm using a microplate reader (Infinite M Plex, Tecan, GER). Each treatment group comprised six replicates, and three independent experiments were performed.

### 2.8 Transwell migration assays

Cell migration assays were carried out in microtiter plates (flat, TC-treated) containing transwell inserts (8 μm pore size, BD Falcon). RAW264.7 cells (3×10^4^) were seeded into the upper chamber in 300 μL serum-free medium, while 800 μL medium containing 20% FBS as chemo-attractant was introduced into the lower chamber. After incubation at 37°C in a 5% CO_2_ atmosphere for 24 h, cells that had migrated through the membrane were fixed with 4% paraformaldehyde for 30 min and subsequently stained with crystal violet for 15 min. Migrated cells were counted in four randomly selected microscopic fields on the Transwell membranes (three replicates) using light microscopy.

### 2.9 Wound healing assay

RAW264.7 cells were plated in 6-well microtiter plates (flat, TC-treated) (4×10^5^ cells/well). After the cells became confluent, the medium was replaced with FBS-free DMEM to block their further proliferation. A sterile 200 μL pipette tip was used to make a scratch in the cell layer in the center of the bottom of the well and then either PBS or designated amounts of EcN-OMVs were added into the medium. Photographs of the bottom of the wells were taken using a light microscope at 0 h and 24 h after scratching the cell monolayer.

### 2.10 Enzyme-linked immunosorbent assay

RAW264.7 cells were plated in 24-well microtiter plates (flat, TC-treated) (1×10^5^ cells/well) and incubated with EcN-OMVs (1.0 μg/mL) or PBS alone for 24 h. Subsequently, culture supernatants and cells were collected, then TNF-α, IL-6, and NO levels in the supernatant were measured with enzyme-linked immunosorbent assays (ELISAs). Intracellular enzymatic activities, including acid phosphatase (ACP), were measured using a microplate reader (Infinite M Plex) according to the manufacturer’s instructions.

### 2.11 ROS level detection

The ROS generation in treated cells with EcN-OMVs was evaluated according to the fluorescence intensity of the 2,7-dichlorofluorescein diacetate (DCFH-DA) probe (Beyotime, Shanghai, China). After treatment, the cells were harvested, washed with PBS, and incubated with the DCFH-DA probe for 30 min at 37°C in the dark. After rinsing, the fluorescent signals were measured at λex = 490 nm/λem = 520 nm using a microplate reader (Infinite M Plex, Tecan, Germany). Additionally, fluorescence images were captured using a fluorescence microscope (TCS SP2, Leica) for qualitative analysis.

### 2.12 RNA sequencing

Total RNA was extracted from macrophages with Trizol Reagent (Ambion, USA), according to the manufacturer’s instructions. Total RNA was analyzed and quantified using an Agilent Bioanalyzer and Qubit (Thermo Fisher Scientific, MA, USA). Sequencing was performed on the Hua Da MGISEQ-T7 platform using the PE150 (paired-end 150) mode. The raw sequencing data underwent quality control using fastp (v2.0) to remove adapters, trim low-quality reads (score < 20), and discard reads with an N content exceeding 10%. The resulting clean reads were mapped to the reference genome using HISAT2 (version 2.1.0), and alignment quality was assessed using RSeQC (v3.0.1). Expression quantification was performed with StringTie. The experiments and sequencing procedures described above were conducted by Chongqing Fulaike Biotechnology Co. Ltd (Chongqing, China) following established protocols.

### 2.13 Gene annotation, identification of differentially expressed genes and statistical analysis

DESeq2 (R packet V 1.24.0) was used to identify DEGs. DEGs were identified by their fold-change in expression value and false discovery rate (FDR) value (*p*adj value, *q*-value or corrected *P* value). DEGs were determined by a |log_2_ fold-change| ≥ 2 and a FDR of less than 0.05. These DEGs were visualized using the ggplot2 package (R version 3.2.1) to create volcano plots. The GO and KEGG enrichment analysis of DEGs was performed using the Fisher precision test (R), and the gene ontology (GO)/Kyoto encyclopedia of genes and genomes (KEGG) terms that were significantly enriched in the DEGs were identified when compared with the whole genomic background.

### 2.14 Real-time quantitative polymerase chain reaction

Total cellular RNA was extracted with Trizol Reagent (Ambion, USA). Two micrograms of total RNA was subjected to reverse transcription with Prime Script RT Master Mix (TaKaRa, Dalian, China). For the reverse transcription of miRNA, a Mir-X miRNA First-Strand Synthesis Kit (TaKaRa) was used. RT-qPCR was conducted with a SYBR Green real-time PCR Master Mix kit (TaKaRa) under these conditions: an initial pre-incubation at 95°C for 30 s, followed by 39 cycles of 95°C for 5 s and 60°C for 30 s. The data were normalized against the expression of β-actin. All data were analyzed using Quant Studio 3 RealTime PCR system software. The forward and reverse primer sequences of target genes are shown in **Table 1**.

**Table 1:** Primer sequences for RT-PCR.

Genes	Primer (5’-3’)	
	Forward	Reverse
Arg1	CTGGGGATTGGCAAGGTGAT	CAGCCCGTCGACATCAAAG
AQP9	TATTGCTCAAGCAGTCCTCAGTC	CCAGAGACACCAAAAGTCGCAT
CD206	TGGTTGGGACTGACCTATGGA	TGGTGGATTGTCTTGTGGAGC
CD86	TTGGGCACAGAGAAACTTGATAG	TTCGGGTGACCTTGCTTAGAC
GAPDH	CCTCGTCCCGTAGACAAAATG	TGAGGTCAATGAAGGGGTCGT
GLUT1	GTGGTGTCGCTGTTTGTTGTAGA	GCACATGCCCACAATGAAGTT
Hk3	CACTGACAGGCACCAAAGAATG	CACATCAATCCTGTAGGTCCCC
ICAM-1	CATCACCGTGTATTCGTTTCCG	TGGCTGGCGGCTCAGTATCT
iNOS	GGGAATCTTGGAGCGAGTTGT	GCACATGCAAGGAAGGGAAC
IL-6	CCCCAATTTCCAATGCTCTCC	CGCACTAGGTTTGCCGAGTA
PFKFB3	AAGAAGCTGACTCGCTACCTC	AGTTGTACTCATTCTCGCCAT
TNF-α	CCCTCACACTCACAAACCACC	CTTTGAGATCCATGCCGTTG
β-actin	GTGACGTTGACATCCGTAAAGA	GTAACAGTCCGCCTAGAAGCAC

### 2.15 Isotope tracer experiment

^13^C-labeled tracer (U-^13^C_6_ glucose) was used to estimate flux through metabolic pathways. Two culture media were used: 1) DMEM medium containing 30% ^13^C_6_-labelled glucose (U-^13^C_6_ glucose); 2) DMEM medium containing 30% ^12^C_6_-labelled glucose. Cell culture and addition of EcN-OMVs were carried out as described in section 2.7.

### 2.16 Intracellular, extracellular, and biomass metabolite extraction

Culture medium (2 mL) from 1 × 10^6^ RAW264.7 cells treated with PBS or EcN-OMVs was used for chemical derivatization of extracellular metabolites. For intracellular metabolite extraction, 4 ml of liquid nitrogen was added to each cell culture dish (60.00mm O.D. x 12.68mm) of RAW264.7 cells. Then cold methanol/sodium hydroxide (1:1), containing the internal standard 2,3,3,3-d4-alanine (0.3 µM), was used to extract metabolites from the RAW264.7 cells. After freezing (-80°C) and thawing (room temperature) samples three times, they were centrifuged at 15,000 g for 15 min at 4°C to separate the supernatant from cellular biomass. The supernatant was retained and stored at -80°C for chemical derivatization of intracellular metabolites. The biomass pellet was resuspended in 200 µL sodium hydroxide and then incubated at 95°C for 1 h. After cooling, 200 µL of methanol was added, and the supernatant was obtained after centrifugation at 15,000 g for 15 min at 4°C, before proceeding with chemical derivatization.

### 2.17 Methyl chloroformate derivatization of metabolites and gas chromatography-mass spectrometry

All prepared extracts were chemically modified by MCF derivatization to lower their boiling point, as described previously (29) The chemical derivatives were analyzed using an Agilent Intu Vo GC9000 gas chromatography (GC) system coupled to an MSD5977A mass spectrometer selective detector (EI) set at 70 eV. A ZB-1701 GC capillary column (20 m × 180 μm × 0.15 mm, Agilent, USA) was used for metabolite analysis. The GC-MS parameters were as previously described (30). The GC-MS inlet was set at 290°C in the pulsed splitless mode and the helium carrier gas flow rate was set to 0.8 mL/min. The temperature was set at 280°C, 230°C, and 150°C for the auxiliary, MS quadrupole, and MS source respectively. The mass range was detected between 38 mm and 550 mm, with a scan speed of 1.562 m/s and the mass spectrometry detector turned on after 6.5 min.

### 2.18 GC-MS processing and statistical analysis

Automated Mass Spectral Deconvolution and Identification System software was used for metabolite deconvolution. The metabolites were identified by mapping the MS fragmentation spectra (mass-to-charge ratio and relative intensity of mass spectra to the highest ion) and corresponding GC retention time to an in-house MCF MS library built using chemical standards. The MassOmics R-based script was used to determine the concentration of the metabolites using the chromatographic height of the most abundant fragmented ion mass. To enhance quantitative robustness and minimize both human and instrumental variability, the relative concentrations of the identified compounds were normalized against internal standards (D4-alanine) and protein concentrations of cells using a BCA protein assay kit. Then, blank samples were used to subtract background contamination and any carryover from identified metabolites. Before analyzing the metabolome, each metabolite concentration was log_10_ transformed and adjusted using Pareto scaling to achieve a Gaussian data distribution. Student’s t-test and FDRs were performed in the R program to determine whether the concentration of each identified metabolite was significantly different between two samples. Principal components analysis (PCA) was conducted to compare intracellular, extracellular, and biomass metabolome profiles between different samples using the MetaboAnalyst 3.0 package for R. Only two-tailed *P*-values less than 0.05 were regarded as statistically significant. Pathway enrichment analysis was performed by blasting our identified metabolites against the KEGG database. The graphical illustrations of heatmaps, line graphs, and chord plots were generated using ggplot2 and GO plot R-packages (31).

### 2.19 Statistical analysis

Data are expressed as mean ± standard deviation (SD) or standard error of the mean (SEM). Statistical analyses were conducted using GraphPad Prism software (version 9, GraphPad Software, USA). The normality of the data distribution was assessed using the Kolmogorov–Smirnov test. For data that did not follow a normal distribution, the Mann-Whitney U test was employed, whereas normally distributed data were analyzed using an unpaired two-tailed t-test for comparisons between two groups. For comparisons involving multiple groups, one-way analysis of variance (ANOVA) followed by Tukey’s *post-hoc* test was applied. A *p*-value less than 0.05 was considered statistically significant. The R code utilized for this study is available in the **Supplementary Material** (R Code).

## 3 Results

### 3.1 Characterization of EcN-OMVs and their uptake by RAW264.7 macrophages

**Figure 1:**
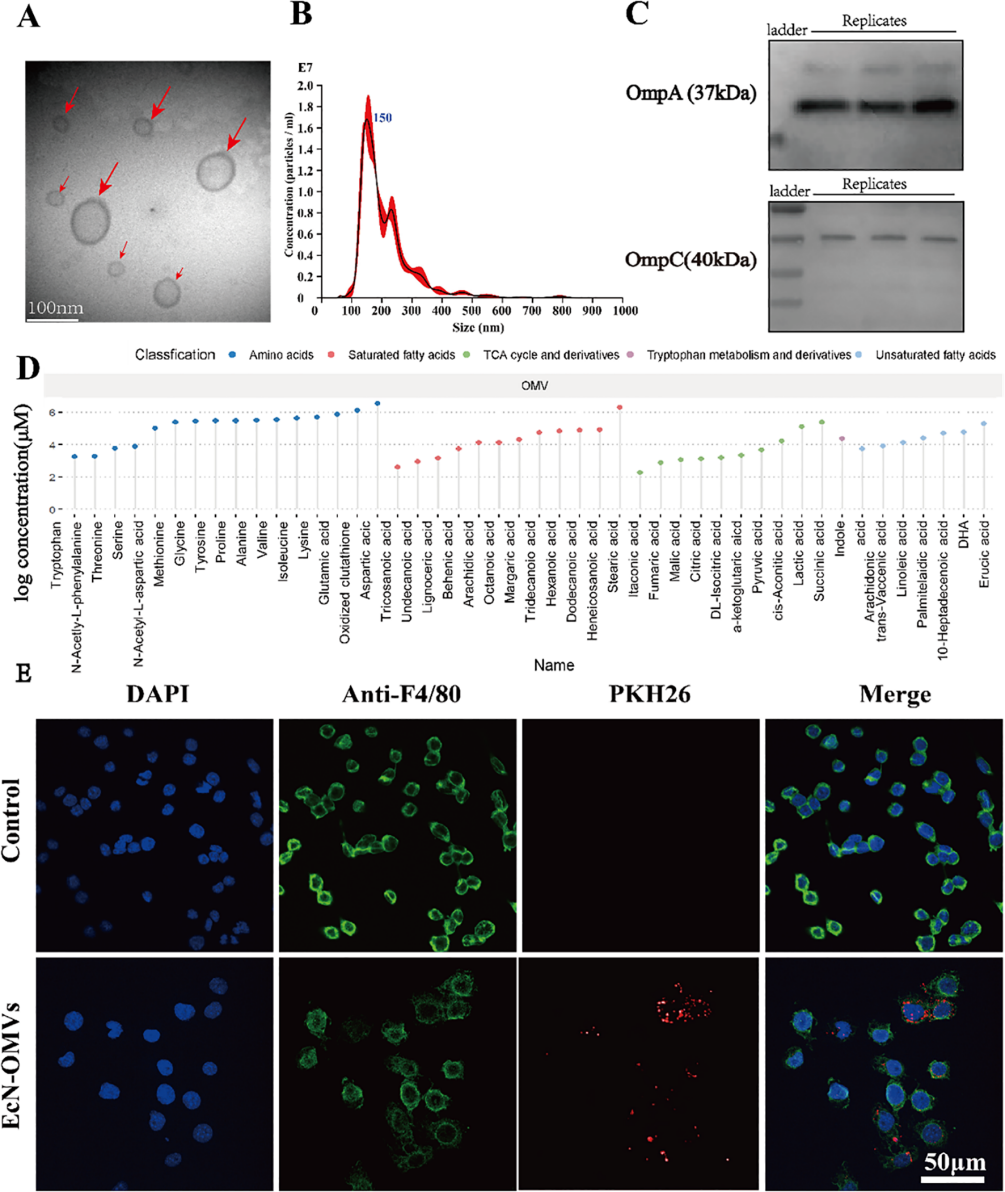
Characterization of EcN-OMVs and their uptake by RAW264.7 macrophages. **(A)** Representative transmission electron microscopy image of OMVs isolated from EcN (indicated by red arrows), bar = 100 nm. **(B)** The size distribution and number of EcN-OMVs determined by nanoparticle tracking analysis. **(C)** Western blot analysis of the expression of EcN-OMV markers OmpA and OmpC. **(D)** Lollipop chart for the metabolome of OMVs. **(E)** Imaging analysis of the uptake of EcN-OMVs by RAW264.7 macrophages. Blue (DAPI), green (Anti-F4/80), and red (PKH26) fluorescent dyes label the nucleus, cell membrane, and EcN-OMVs respectively.

The quality and purity of the obtained EcN-OMVs were assessed. Transmission electron microscopy demonstrated that the vesicles were spherical particles with a size range of 50–200 nm (**Figure 1A**). Nanoparticle tracking analysis showed a peak at 150 nm for the size distribution of these vesicles (**Figure 1B**). Western Blot analysis confirmed expression of vesicle marker proteins OmpA and OmpC in EcN-OMVs (**Figure 1C**). A total of forty-six metabolites were identified and quantified in OMVs, which comprised sixteen amino acids, ten saturated fatty acids, seven unsaturated fatty acids, twelve TCA cycle derivatives, and one tryptophan derivative (**Figure 1D**).

To determine whether the EcN-OMVs could be taken up by macrophages, EcN-OMVs were labelled with the red fluorescent dye PKH26. After co-culture of the labeled EcN-OMVs with macrophages for 24 h, EcN-OMVs were observed in the cytoplasm of the macrophage, indicating that these vesicles were internalized by RAW264.7 cells (**Figure 1E**). In addition, EcN-OMV treatment did not induce apoptosis in RAW264.7 macrophages, as shown in **Supplementary Figure 1**.

### 3.2 EcN-OMVs promote macrophage function and polarization toward an M1-like phenotype

**Figure 2:**
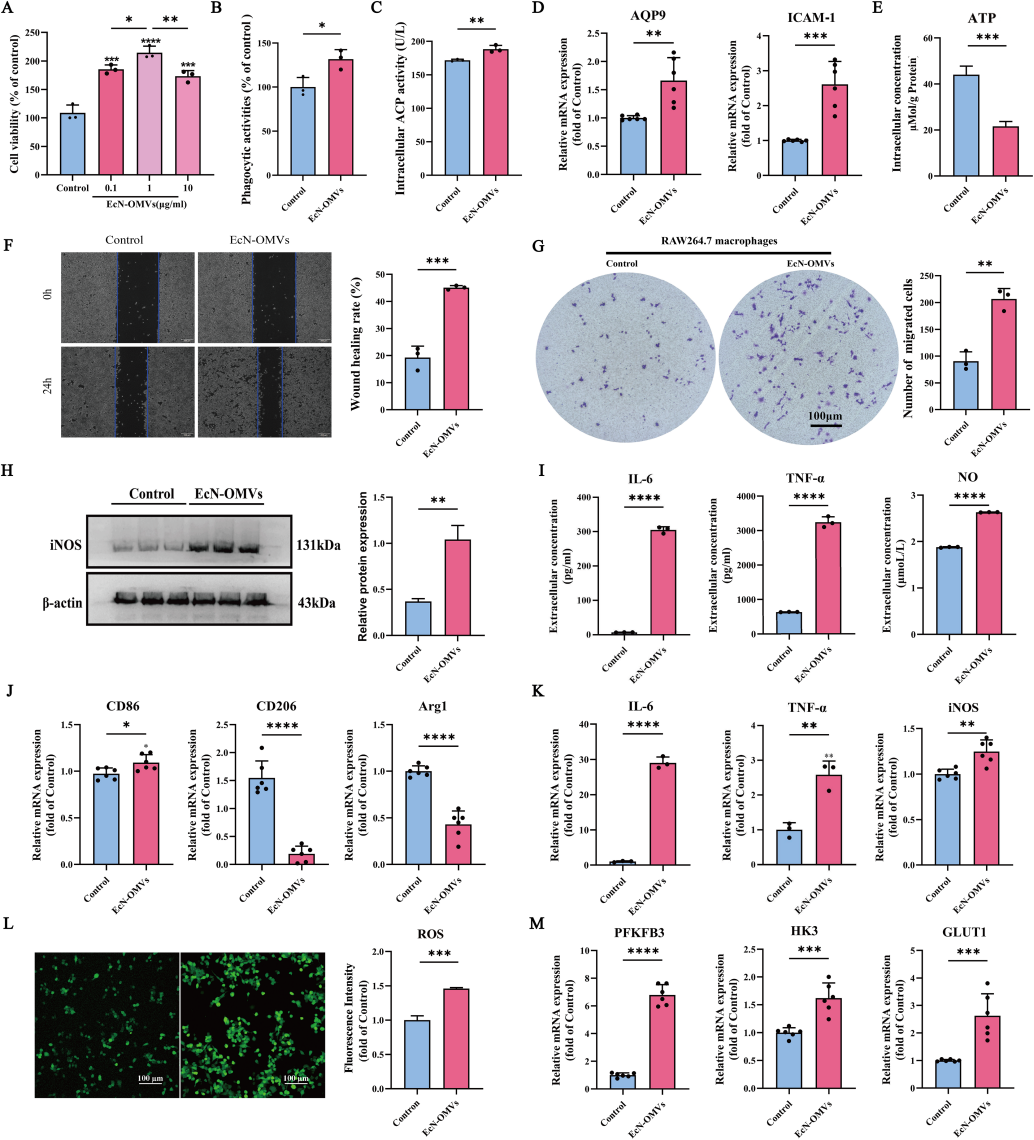
EcN-OMVs promote the proliferation, phagocytic activity, migration, and M1 polarization of RAW264.7 macrophages. **(A)** Viability of RAW264.7 cells 24 h after exposure to EcN-OMVs at 0.1 µg/mL, 1 µg/mL, and 10 µg/mL. **(B)** Phagocytic activity of RAW264.7 cells 24 h after stimulation with EcN-OMVs. **(C)** Acid phosphatase (ACP) activities in macrophage lysates 24 h after stimulation with EcN-OMVs. **(D)** mRNA expression of AQP9 and ICAM-1 in macrophages determined by qPCR. **(E)** EcN-OMVs reduce intracellular ATP levels. **(F)** Wound healing migration of macrophages after co-culture with EcN-OMVs, bar = 200 μm. **(G)** The migration of macrophages after co-culture with EcN-OMVs determined using transwell microtiter plates. **(H)** Western blot analysis of iNOS (M1 macrophage marker) expression in macrophages relative to expression of ß-actin. **(I)** Expression of TNF-α, IL-6, and NO in macrophages exposed to PBS or EcN-OMVs determined using ELISAs. **(J)** mRNA expression of CD86, CD206, and Arg1 in macrophages determined by qPCR. **(K)** mRNA expression of IL-6, TNF-α, and iNOS in macrophages determined by qPCR. **(L)** EcN-OMVs induce ROS accumulation. Intracellular ROS was measured using a fluorescence microscope and fluorescence intensity at λex490 nm/λem520 nm with a microplate reader, bar = 200μm. **(M)** mRNA expression of PFKB3, HK3, and GLUT1 in macrophages determined by qPCR. Results are presented as means ± SEM, n=3, *P < 0.05; **P < 0.01; ***P < 0.001; ****P < 0.0001.

To explore the effects of EcN-OMVs on macrophage function, RAW264.7 cells were incubated with various concentrations of EcN-OMVs (0.1 µg/mL, 1 µg/mL, or 10 µg/mL) for 24 h, and changes in proliferation were measured using a cell viability assay. As shown in **Figure 2A**, the cell viability was increased by 85.5%, 114.68%, and 73.52% after exposure to 0.1 µg/mL, 1 µg/mL, and 10 µg/mL of EcN-OMVs, respectively. As the most pronounced increase was observed at 1 µg/ml, this concentration of EcN-OMVs was used for subsequent experiments.

To investigate phagocytic activity, RAW264.7 cells were incubated with EcN-OMVs and neutral red. Phagocytic activity increased significantly after EcN-OMV stimulation (**Figure 2B**). In addition, ACP activity, involved in the degradation of phagocytosed material, was enhanced in macrophages co-cultured with EcN-OMVs (**Figure 2C**). RT-qPCR analysis revealed that the expression of phagocytosis related molecules AQP9 and ICAM-1 were upregulated in the EcN-OMV-treated group (**Figure 2D**). This suggests that EcN-OMVs stimulate the phagocytic function of RAW264.7 macrophages.

The effect of EcN-OMVs on macrophage migration was evaluated using wound healing and transwell migration assays. The wound closure rate of macrophages treated with OMVs was 2.36-fold greater at 24 h than PBS-treated controls (**Figure 2F**). Similarly, in the transwell migration assay, macrophages treated with EcN-OMVs displayed a higher migration rate than controls (**Figure 2G**). These results demonstrated that EcN-OMVs promote macrophage migration.

In addition, the polarization effect of EcN-OMVs on macrophages was evaluated. After treatment with 1 µg/ml EcN-OMVs for 24 h, RAW264.7 cells showed significant increases in the levels of M1-associated proinflammatory cytokines, including TNF-α, IL-6, and NO, as measured by ELISAs (**Figure 2I**). The expression of iNOS, an enzyme responsible for NO synthesis, was also upregulated (**Figure 2K**). EcN-OMVs also induced a pronounced shift in macrophage polarization, characterized by marked upregulation of proinflammatory M1 markers (CD86, IL-6, and TNF-α) and suppression of anti-inflammatory M2 markers (CD206 and Arg1) revealed by RT-qPCR analysis (**Figures 2J, K**). Moreover, EcN-OMV treatment triggered metabolic reprogramming in RAW264.7 macrophages, with intracellular ROS levels markedly elevated (**Figure 2L**) and ATP production significantly suppressed (**Figure 2E**) relative to controls. Collectively, these findings demonstrate that EcN-OMVs drive macrophages toward an M1-like phenotype through coordinated transcriptional and metabolic alterations, favoring a proinflammatory state.

### 3.4 Metabolite profiling of intracellular, extracellular, and biomass samples from macrophages treated with EcN-OMVs

**Figure 3:**
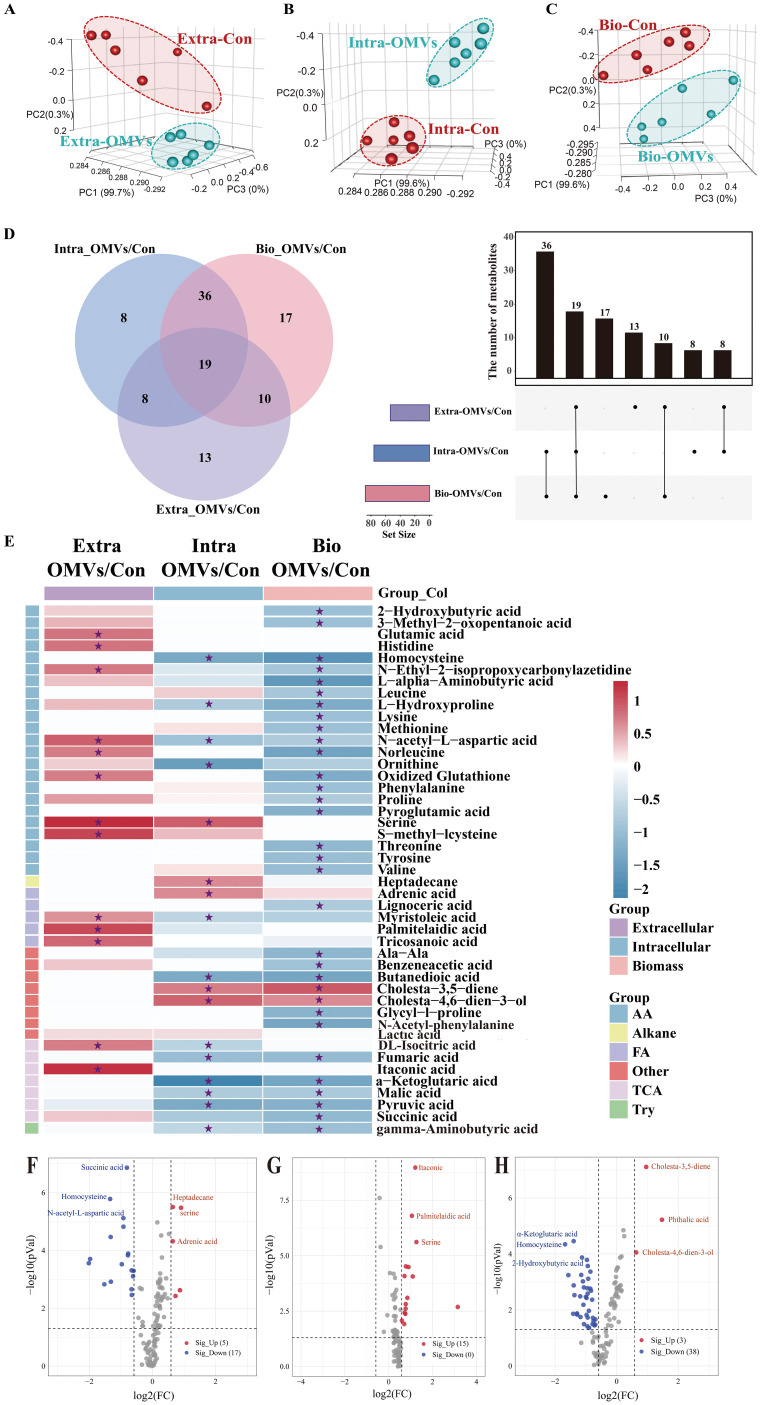
Metabolomic profiling of macrophages. Principal component analysis (PCA) of the metabolite profiles of extracellular **(A)**, intracellular **(B)**, and biomass **(C)** macrophage samples. Red dots indicate the control group (RAW264.7 cells), while green dots represent samples treated with EcN-OMVs. Each group comprised six replicates. **(D)** Venn diagram and upset plot showing metabolites with significantly different concentrations between groups (p < 0.05). The dots and connected bars represent the number of metabolites with altered concentrations, either unique to, or shared among, macrophage samples. **(E)** Heatmap illustrating metabolite profiles of the three sample types and their corresponding metabolic classifications. Red blocks indicate higher metabolite concentrations in the EcN-OMV group compared to the control, while blue blocks indicate lower concentrations. Relative metabolite concentrations are shown on a log_2_ scale. Only metabolites with p-values < 0.05 are displayed, and those with FDR (q-values) < 0.05 are labeled with asterisks. **(F–H)** Volcano plots showing metabolites with significantly altered concentrations between groups (p < 0.05, FC > 1.5) in intracellular **(F)**, extracellular **(G)**, and biomass **(H)** macrophage samples. Red dots represent upregulated metabolites, while blue dots represent downregulated metabolites.

GC-MS-based metabolic profiling identified over 150 chromatographic peaks, including 120, 87, and 120 metabolites in intracellular, extracellular, and biomass samples, respectively. 3D-PCA showed clear segregation between the EcN-OMV and control groups across all three sample types (intracellular, extracellular, and biomass; **Figures 3A-C**).

The Venn diagram and Upset plot in **Figure 3D** indicate the numbers of common and unique metabolites with significantly different concentrations between groups for the three sample types. Concentrations of 27 (8 + 19) metabolites were significantly altered in both intracellular and extracellular samples between the control and EcN-OMV groups. The intracellular and extracellular samples shared 55 (36 + 19) and 29 (19 + 10) metabolites, respectively, with the biomass samples. A total of 8, 13, and 17 unique metabolites showed significantly altered concentrations between groups for the intracellular, extracellular, and biomass samples, respectively. In addition, 19 metabolites exhibited significant concentration differences across all three sample types. Heatmap and volcano plots (**Figures 3E–H**) depicted the relative increases and decreases in metabolite concentrations following EcN-OMV treatment. A total of 111 metabolites showed significant alterations in concentration between macrophages treated with EcN-OMVs and the control group across the three sample types, as illustrated in the heatmap (**Figure 3E**, p < 0.05, *q* < 0.05). Significantly altered metabolites due to EcN-OMV treatment were classified as amino acids and derivatives, alkanes, fatty acids, TCA cycle intermediates, and other organic acids. Most extracellular metabolites had higher relative concentrations in the EcN-OMV group compared to controls, whereas significantly decreased concentrations were primarily observed in the biomass samples.

To be more specific, the extracellular concentrations of itaconic acid, DL-isocitric acid, serine, S-methyl-L-cysteine, N-acetyl-L-aspartic acid, glutamic acid, histidine, tryptophan, norleucine, oxidized glutathione, palmitelaidic acid, tricosanoic acid, and myristoleic acid were significantly increased by EcN-OMV treatment, while that treatment resulted in no significant decreases in extracellular metabolite concentrations (**Figure 3G**, FC > 1.5, *p* < 0.05, *q* < 0.05). Furthermore, the increased extracellular levels of amino acids such as serine, glutamate and histidine may reflect enhanced amino acid secretion from macrophages induced by EcN-OMVs. The intracellular concentrations of heptadecane, serine, cholesta-4,6-dien-3-ol, adrenic acid, and cholesta-3,5-diene increased significantly in the EcN-OMV group compared to controls. In contrast, α-ketoglutaric acid, ornithine, homocysteine, butanedioic acid, pyruvic acid, fumaric acid, N-acetyl-L-aspartic acid, succinic acid, malic acid, L-hydroxyproline, DL-isocitric acid, myristic acid, and gamma-aminobutyric acid showed significantly decreased concentrations in the EcN-OMV group (**Figure 3F**, FC > 1.5, *p* < 0.05, *q* < 0.05). The reductions in intracellular succinic acid, malic acid, and fumaric acid may be related to a shift in central carbon metabolism, likely associated macrophage activation. In the biomass samples the concentrations of phthalic acid, cholesta-3,5-diene, and cholesta-4,6-dien-3-ol were significantly increased by EcN-OMV treatment, while those of succinic acid, malic acid, pyruvic acid, DL-isocitric acid, α-ketoglutaric acid, fumaric acid, N-acetyl-L-aspartic acid, proline, serine, leucine, lysine, methionine, phenylalanine, 2-hydroxybutyric acid, valine, tyrosine, tryptophan, threonine, pyroglutamic acid, L-hydroxyproline, oxidized glutathione, norleucine, L-alpha-aminobutyric acid, homocysteine, and lignoceric acid were significantly decreased (**Figure 3H**, FC > 1.5, *p* < 0.05, *q* < 0.05). Meanwhile, elevated lipid levels and reduced levels of amino acids and TCA cycle intermediates in macrophage biomass suggest a shift in resource allocation to support alternative biosynthetic processes in response to EcN-OMVs.

### 3.5 EcN-OMVs modulate macrophage metabolic pathways

**Figure 4:**
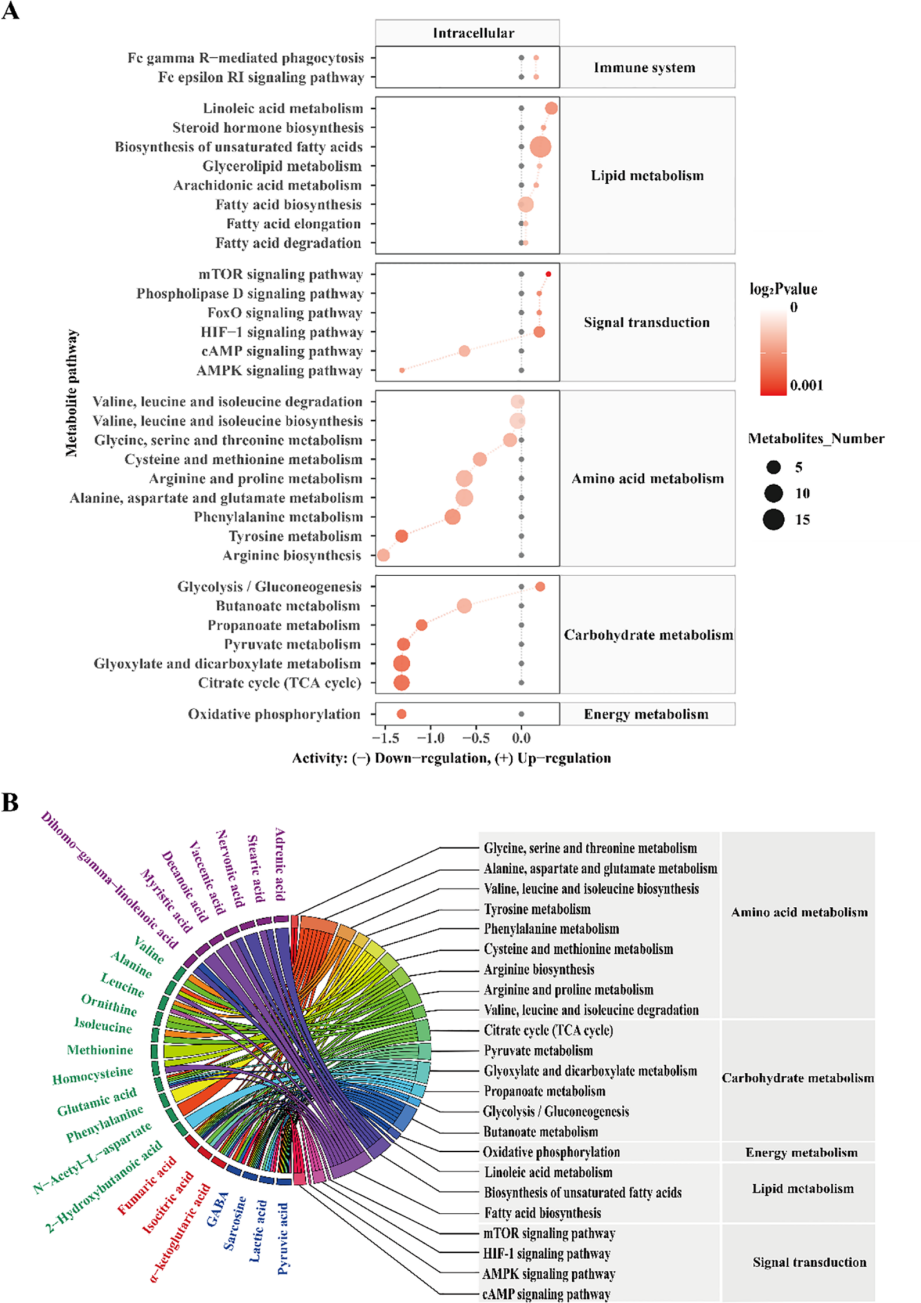
Metabolic pathway enrichment analysis **(A)**. Black dots represent metabolic activities in the control group that were adjusted to 0. Red dots represent metabolic activities in the EcN-OMV group compared to the control group. The dot size represents the number of metabolites in a pathway, and the intensity of the red color indicates the p-value significance. The predicted metabolic activities were visualized using a log_2_ scale. **(B)** A chord plot displaying how the metabolites link to different pathways. Metabolite classifications were defined by the following colors (purple = fatty acids, green = amino acids and their derivatives, red = TCA cycle intermediates, blue = organic acids).

To further explore how EcN-OMVs affect the metabolic status of polarized macrophages, we performed pathway enrichment analysis using the KEGG metabolic network (**Figure 4A**). The analysis revealed that EcN-OMVs significantly altered key metabolic pathways related to macrophage polarization. Specifically, pathways associated with cell growth and death, the immune system, lipid metabolism, and signal transduction were upregulated in the EcN-OMV group, except for the cyclic adenosine monophosphate (cAMP) and AMPK signaling pathways. In contrast, pathways related to amino acid metabolism, carbohydrate metabolism (except for glycolysis and gluconeogenesis), and energy metabolism were downregulated. These results suggest that EcN-OMVs reprogram macrophage metabolism by selectively modulating key pathways involved in energy production and immune responses. Further analysis identified that alanine, aspartate, and glutamate metabolism, as well as fatty acid biosynthesis, were linked to the highest number of significantly altered metabolites, as indicated in the chord plot (**Figure 4B**). Metabolites such as pyruvic acid and lactic acid were significantly enriched in the glycolytic pathway, reflecting a shift towards glycolysis in macrophages treated with EcN-OMVs. In addition, we assessed the expression levels of the key glycolytic enzymes glucose transporter 1 (GLUT1), hexokinase 3 (HK3) and 6-Phosphofructo-2-Kinase (PFKFB3), and found they were significantly upregulated by EcN-OMV treatment, which is consistent with the activation of the glycolytic pathway (**Figure 2M**). Similarly, the TCA cycle was linked to key intermediates, including fumaric acid, isocitric acid, and α-ketoglutaric acid, which are critical for energy production and biosynthesis during macrophage activation. Notably, oxidative phosphorylation, another major energy pathway, showed changes in metabolites such as sarcosine and fumaric acid, indicating altered mitochondrial function.

These metabolic pathways are closely associated with macrophage polarization (32). For instance, increased glycolysis (33), as indicated by elevated pyruvic and lactic acid levels, is a hallmark of M1 macrophage polarization, while the TCA cycle and oxidative phosphorylation contribute to M2 polarization by supporting anabolic processes and energy production. Furthermore, pathways such as mammalian target of rapamycin (mTOR), hypoxia-inducible factor 1 (HIF-1), and 5’ AMP-activated protein kinase (AMPK) signaling, which were identified in our analysis, are well-known regulators of macrophage metabolic reprogramming and polarization (34, 35). Together, these findings suggest that EcN-OMVs promote macrophage polarization by modulating key metabolic pathways, including glycolysis, the TCA cycle, and oxidative phosphorylation.

### 3.6 Transcriptomic profiling of the response of macrophages to EcN-OMVs

**Figure 5:**
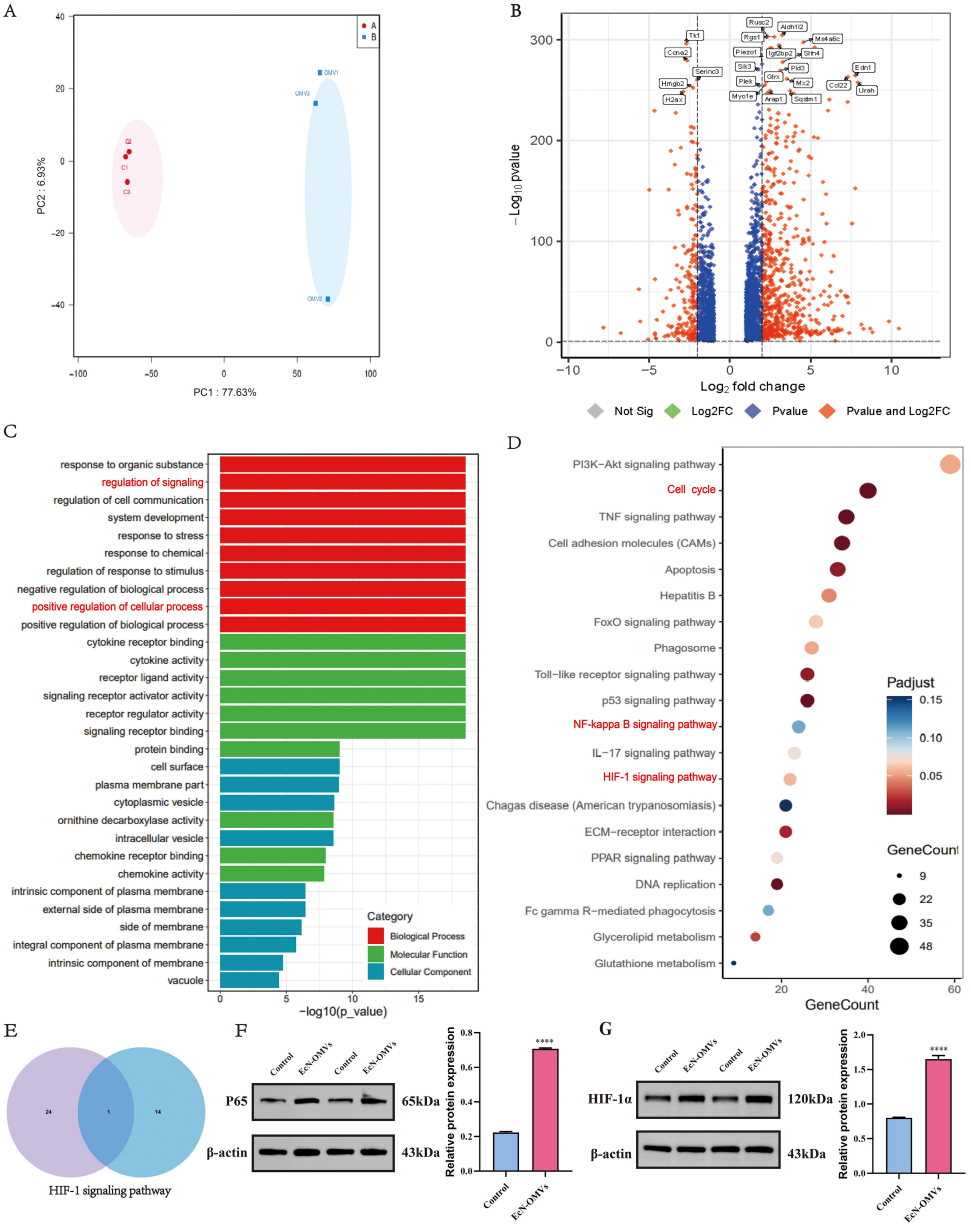
Transcriptomic analysis of the response of macrophages to EcN-OMVs. **(A)** Principal component analysis (PCA) of the RNA-sequencing (RNA-Seq) data comparing the EcN-OMV treatment (blue) and control (red) groups. **(B)** Volcano plot of the RNA-Seq data. The red and blue data points represent the upregulated genes (log2FC > 2, FDR < 0.05) and downregulated genes (log2FC < -2, FDR < 0.05), respectively, in response to EcN-OMV treatment. **(C)** GO classification analysis of differentially expressed genes (DEGs) in level two. **(D)**. KEGG classification analysis of the top 20 DEGs. The dot size represents the number of genes in a pathway, and the intensity of the color indicates the p-value significance. **(E)** Venn diagram showing the overlap between the metabolome KEGG enrichment (left, top 25 metabolites) and the transcriptome KEGG enrichment (right, top 15 genes). **(F, G)** Western blot analysis of P65 and HIF-1α expression in macrophages, normalized to β-actin. Results are presented as means ± SEM, n = 3, *P < 0.05; **P < 0.01; ***P < 0.001; ****P < 0.0001.

To confirm the effect of EcN-OMVs on macrophages, a transcriptomic analysis was performed to determine changes at the gene expression level. PCA displayed a clear differentiation between control and EcN-OMV-treated macrophages (**Figure 5A**). A total of 877 DEGs (FC > 4 and adjusted *p*-value < 0.05) were identified, of which 214 were downregulated and 663 were upregulated in response to EcN-OMVs (**Figure 5B**). By analyzing these DEGs and their functions, we gained a deeper understanding of the role of EcN-OMVs in promoting macrophage activity. Using the GO classification, the DEGs were assigned to 52 categories. In the category of ‘biological process’ (**Figure 5C** red horizontal bars), upregulated DEGs were classified into ‘regulation of signaling’ (99 genes), ‘response to organic substance’ (98 genes), ‘regulation of cell communication’ (98 genes), and ‘system development’ (96 genes). The molecular function category (**Figure 5C**) showed that ‘signaling receptor binding’ (96 genes), ‘signaling receptor activator activity’ (96 genes) and ‘receptor ligand activity’ (48 genes) obviously affected major processes in the response of macrophages to EcN-OMVs. In the category of cellular components, upregulated DEGs were classified into ‘cell surface’ (50 genes), ‘plasma membrane part’ (102 genes), and ‘cytoplasmic vesicle’ (84 genes) (**Figure 5C**). The KEGG pathways enrichment showed that the significantly enriched pathways involving the upregulated genes included DNA replication, the cell cycle, and the TNF signaling pathway (**Figure 5D**). Interestingly, HIF-1α emerged as the sole common signaling pathway linking transcriptomic and metabolomic reprogramming in macrophages, as evidenced by Venn diagram intersection (**Figure 5E**). To functionally validate these findings, we confirmed elevated protein levels of HIF-1α and NF-κB p65 in EcN-OMV-treated macrophages using Western blot analysis (**Figures 5F, G**). This concordance between multi-omics profiling and protein-level validation underscores HIF-1α as a central mediator of EcN-OMV-induced macrophage polarization.

### 3.7 Isotopically-labeled glucose tracing of extracellular, intracellular, and biomass metabolites

**Figure 6:**
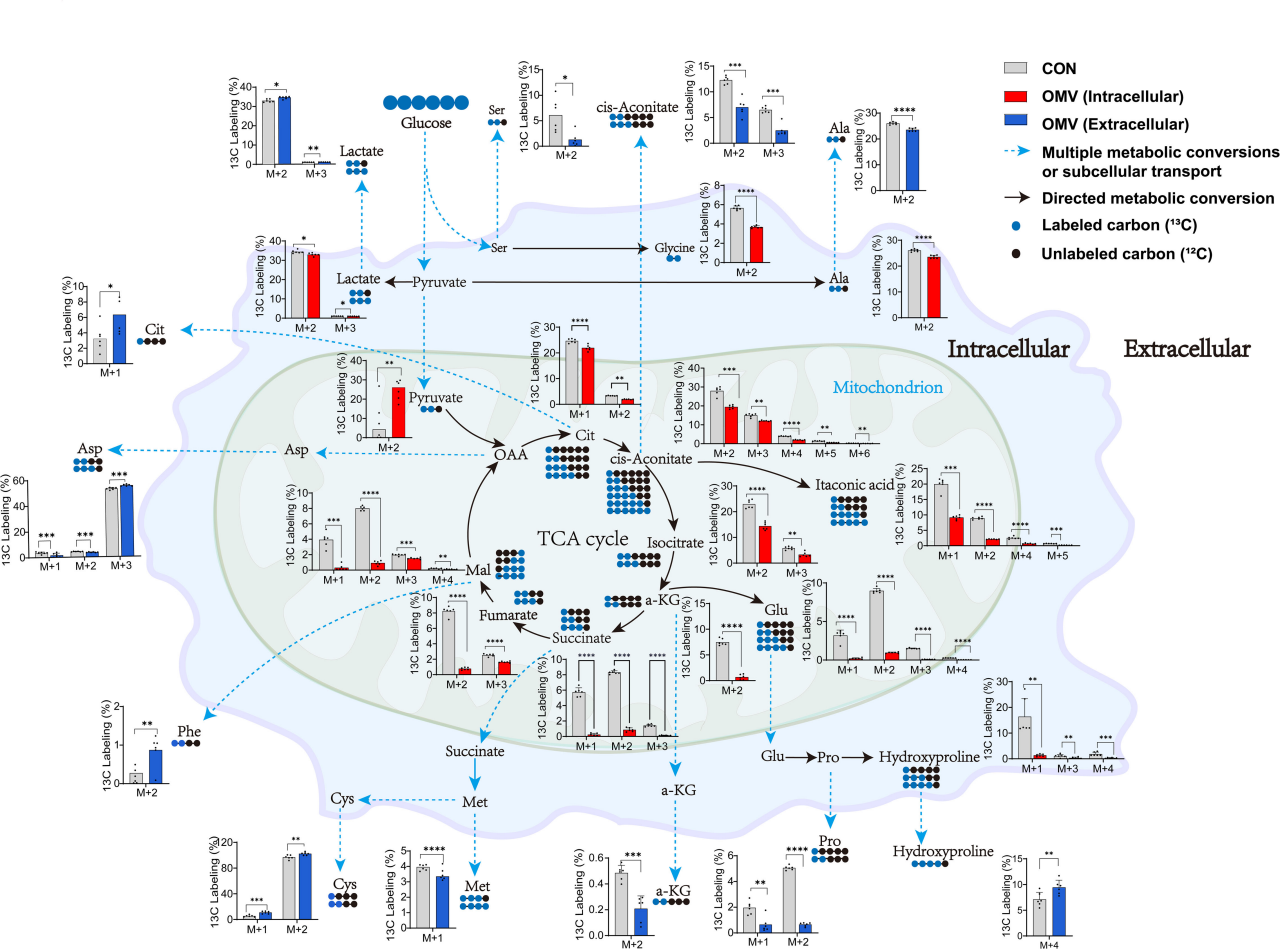
Incorporation of ^13^C derived from ^13^C-labelled glucose into intracellular and extracellular metabolites in RAW264.7 cells with (red/blue) or without (grey) EcN-OMV treatment. The primary molecular ion of the identified metabolites is denoted as M, while the M+n ion exhibits a mass-to-charge ratio that is n units higher than that of the M ion. This means that the compound has n ^13^C atom labels. Circles indicate the number of labelled carbon-13 (blue) and cabon-12 (black) atoms in a metabolite. For larger versions of the bar charts, see **Supplementary Figures 2** and **3**. The y-axis shows the relative percentage of ¹³C labeling in M+n isotopologues compared to the corresponding unlabeled molecular ion (M) of the same metabolite. Statistical significance was calculated using the student’s t-test: *p < 0.05; **p < 0.01; ***p < 0.001; ****p < 0.0001.

To track how the EcN-OMVs affect metabolic flux in macrophages, we supplied RAW264.7 macrophages with 30% ^13^C-labelled glucose and followed its metabolic conversions within extracellular, intracellular, and biomass samples. Our premise was that a higher rate of biochemical conversion is demonstrated when the proportion of ^13^C-labelled carbon within a metabolite is elevated, indicating that carbon skeletons of glucose are fluxed into the labelled metabolites (**Figure 6**).

We observed a significant decrease in ^13^C-labelling for seven extracellular metabolites in the culture medium of macrophages treated with EcN-OMVs. These metabolites were alanine (M+2), aspartic acid (M+1-2), methionine (M+1), proline (M+1-2), serine (M+2), cis-aconitic acid (M+2-3), and α-ketoglutaric acid (M+2). Conversely, seven metabolites had significantly increased ^13^C-labelling, they were aspartic acid (M+3), L-hydroxyproline (M+4), phenylalanine (M+2), S-methyl-L-cysteine (M+1-2), stearic acid (M+8), citric acid (M+1), and lactic acid (M+2-3) (**Supplementary Figure 2**).

The intracellular metabolite profiles demonstrated significant fluctuations in the ^13^C-labelling of TCA cycle intermediates in the macrophages co-cultured with EcN-OMVs. α-Ketoglutaric acid (M+2), cis-aconitic acid (M+2-6), citric acid (M+1-3), isocitric acid (M+2-4), fumaric acid (M+3-4), itaconic acid (M+1-2, 4-5), lactate (M+2-3), malate (M+1-4) and succinic acid (M+2) had reduced ^13^C-labelling, with pyruvate (M+2) being the only intermediate showing an increase. A trend of decreased ^13^C-labelling was observed for fatty acids and their derivatives, including 11,14,17-eicosatrienoic acid (M+18), arachidic acid (M+2,4,6), behenic acid (M+2), dodecanoic acid (M+4-5), homo-γ-linolenic acid (M+6), lignoceric acid (M+2), linoleic acid (M+6), myristic acid (M+8), palmitelaidic acid (M+2,4), and stearic acid (M+1-10). Amino acid labelling was also decreased for alanine (M+2), glutamic acid (M+1-4), glycine (M+2), and L-hydroxyproline (M+1,3,4) (**Supplementary Figure 3**).

We observed a significant decrease in ^13^C-labelling of 30 metabolites in the biomass of macrophages co-cultured with EcN-OMVs. These included 2-hydroxybutyric acid (M+1), alanine (M+2), glycine (M+2), histidine (M+4), N-acetyl-L-aspartic acid (M+4), oxidized glutathione (M+4), proline (M+2,3,4), and serine (M+1,2), however homocysteine (M+3), showed elevated ^13^C labelling. Meanwhile, there was a significant reduction in ^13^C-labelling of most TCA cycle intermediates and fatty acids. Specifically, α-ketoglutaric acid (M+2), cis-aconitic acid (M+2,3), citric acid (M+2,3), DL-isocitric acid (M+1-4), fumarate (M+2,3), itaconic acid (M+1,2,4), lactic acid (M+2,3), malic acid (M+2), pyruvic acid (M+2), succinic acid (M+2,3), 11,14,17-eicosatrienoic acid (M+6,7,8,17), 11,14-eicosadienoic acid (M+4,6,10), arachidic acid (M+10-16), behenic acid (M+2,3,4,5,6,8), dodecanoic acid (M+2,3,4), erucic acid (M+2-5), γ-linolenic acid (M+6,7,8,10), hexanoic acid (M+2), homo-γ-linolenic acid (M+4,6,12) and lignoceric acid (M+2-10) showed reduced ^13^C labeling, while only GABA (M+3) displayed an increased ^13^C labelling (**Supplementary Figure 5**).

## 4 Discussion

Our study is the first to integrate metabolomic profiling, transcriptomic analysis, and isotope labelling experiments to explore the metabolic effects of EcN-OMVs on the regulation of macrophage proliferation, migration, phagocytosis, and polarization. We found that EcN-OMVs promoted the proliferation, migration, phagocytosis, and M1 polarization of RAW264.7 macrophages. These phenotypic phenomena were also accompanied by elevated glycolytic flux, suppressed TCA cycle activity, and accumulation of extracellular nutrients such as amino acids and fatty acids. These results increase our understanding of the probiotic effect of OMVs from *E. coli* Nissle 1917 on macrophage behavior and metabolic reprogramming.

### 4.1 EcN-OMVs induce M1 polarization of RAW264.7 macrophages

Previous studies have reported that EcN-OMVs are immunobiologically active and capable of modulating the inflammatory response of macrophages (27). Macrophages are highly adaptable cells capable of quickly altering their functional profiles through a process known as polarization. Although no prior investigation has reported the influence of EcN-OMVs on macrophage polarization, our results revealed that internalization of EcN-OMVs led to phenotypic changes characteristic of M1-type polarization in RAW264.7 macrophages. Our findings showed that EcN-OMVs induced RAW264.7 macrophages to secrete the proinflammatory cytokines TNF-α, IL-6, and NO (**Figure 2I**), and enhanced the transcription of genes encoding TNF-α, and IL-6 (**Figure 2K**). In addition, EcN-OMV treatment led to increased expression of M1 markers (CD86, IL-6, and TNF-α) and decreased expression of M2 markers (Arg1 and CD206) (**Figures 2J, K**). Notably, our findings are similar to those in recent studies on bone marrow-derived macrophages which found that OMVs induced an increase in the expression of inflammatory markers in macrophages (36, 37). Toh et al. demonstrated that M1 macrophages secreted proinflammatory cytokines such as IL-1b, TNF-α, and chemokines (38). Similarly, Covarrubias et al. showed that M1 polarization in monocyte-derived human macrophages (MDMs) was associated with the production of ROS and the secretion of NO (39). In addition, Gordon and Martinez reported that M1 macrophages generated a higher concentration of key inflammatory cytokines, such as IL-12 and IL-6 (40). Moreover, our results revealed that EcN-OMVs promoted the phagocytosis, proliferation, and migration of RAW264.7 macrophages (**Figure 2**). Collectively, these findings indicated that EcN-OMVs exert immunomodulatory effects on macrophages and induce M1-polarization in RAW264.7 macrophages.

### 4.2 Metabolome of OMVs associated with macrophage M1 polarization

To further explore the potential mechanism by which EcN-OMVs affect macrophage polarization, we performed a quantitative metabolomics analysis of EcN-OMVs. Among the 20 metabolites with the highest concentrations, were stearic acid, isoleucine, valine, succinic acid, and heneicosanoic acid, which are all known to induce M1 polarization. (**Figure 1D**, **Supplementary Table 1**). Saturated fatty acids such as stearic acid can induce inflammation through signaling pathways involving toll-like receptors (TLRs). Exposure to stearic acid promotes a metabolic shift in macrophages toward glycolysis (41). Similarly, branched-chain amino acids (BCAAs), including isoleucine and valine, support acetylation and provide acetyl CoA derivatives, potentially activating mTORC1 and further enhancing glycolysis (42). Furthermore, succinate has been implicated in regulating macrophage polarization through its role in modulating the inflammatory response via succinate receptors on internal organs and immune cells (16). Succinate is a product of the reaction catalyzed by proline hydroxylase using alpha-ketoglutarate as a substrate, and can feedback-inhibit the reaction. Acting as the substrate for Complex II of the mitochondrial respiratory chain, succinate drives ROS production in macrophages (43), and through reverse electron transport, succinate can fuel ROS production by Complex I. Moreover, succinate stabilizes HIF-1α by inhibiting proline hydroxylase activity and enhancing ROS production in M1-macrophages (17). Previous studies have indicated that the HIF-1α signaling pathway can upregulate GLUT1, HK3, PFKFB3, and other glycolytic-related proteins, thereby increasing glycolysis (44, 45). Importantly, we found mRNA expression levels of GLUT1, HK3, and PFKFB3 were significantly upregulated in EcN-OMV-treated macrophages (**Figure 2M**). In addition, both metabolomic and transcriptomic KEGG enrichment analyses revealed significant upregulation of the HIF-1 signaling pathway in EcN-OMV-treated macrophages (**Figures 4A**, **5D**). Western blot analysis further confirmed a significant increase in HIF-1α protein levels (**Figure 5G**). Thus, succinate in EcN-OMVs may act as a key metabolite regulating macrophage polarization via the HIF-1α signaling pathway. However, EcN-OMVs also contain high levels of anti-inflammatory metabolites such as α-ketoglutaric acid, glutamate, and glutathione. The mechanism by which these metabolites from EcN-OMVs promote macrophage polarization remains to be investigated.

### 4.3 EcN-OMVs induce glycolysis and suppress the TCA cycle in RAW264.7 macrophages

Previous studies have demonstrated that the polarization of macrophages is often accompanied by increased anaerobic respiration (46). Our findings revealed that both intracellular and extracellular lactic acid concentrations in macrophages increased in response to EcN-OMVs (**Figure 3E**). Isotopic tracer experiments showed a significant increase in ^13^C-labeled lactic acid (M+2-3) (**Figure 6**), which is a product of glycolysis. The elevated lactic acid levels indicate an upregulation of glycolytic activity in macrophages. Proinflammatory macrophages (M1) typically exhibit reduced mitochondrial activity alongside a substantial increase in glycolytic metabolism (46), crucial for generating the ATP needed to sustain phagocytosis (47). Glycolysis also supports the pentose phosphate pathway (PPP), promoting the production of nicotinamide adenine dinucleotide phosphate (NADPH) and ribose-5-phosphate. NADPH is involved in cellular ROS production and plays a crucial role in macrophage phagocytosis (48). Ribose-5-phosphate serves as a precursor to nucleotides and amino acids that are essential for cell growth and proliferation, which is consistent with our observation that EcN-OMVs promote macrophage proliferation and migration (**Figures 2F, G**). Furthermore, our results indicate that EcN-OMVs not only upregulate macrophage glycolysis but also downregulate the TCA cycle. We observed lower concentrations of all TCA cycle intermediates, including succinic acid and α-ketoglutaric acid (**Figures 3E-H**), and reduced ^13^C-labeled glucose flux through the cycle in cells treated with EcN-OMVs (**Supplementary Figure 2**). Studies have shown that itaconic acid can promote the expression of IL-6 by increasing the protein level of transcriptional activator 3 (ATF3) (49). Our results showed the extracellular accumulation of itaconic acid (**Figure 3G**). Thus, we propose that OMVs suppress the conversion of citric acid to α-ketoglutaric acid in macrophages, leading to itaconic acid production, which may stimulate ATF3 expression and promote IL-6 secretion (**Figure 2I**). These metabolic alterations notably reflect the M1 macrophage phenotype. Previous studies have reported enhanced glycolysis and decreased oxygen consumption in M1 macrophages (50, 51), while Rodríguez-Prados et al. noted reduced mitochondrial oxidative phosphorylation in M1 macrophages compared to M0 macrophages (52). Thus, treatment with EcN-OMVs suppresses aerobic respiration in macrophages. Indeed, M1 macrophages primarily rely on glycolysis as their main energy source, exhibiting reduced dependence on oxidative phosphorylation (OXPHOS) in mitochondria (53). This metabolic shift to glycolysis is closely associated with alterations in mitochondrial dynamics, specifically favoring fission over fusion (54, 55). Increased mitochondrial fission and decreased fusion results in mitochondrial fragmentation (56), which enables the metabolic reprogramming necessary for rapid glycolytic ATP production but sacrifices overall ATP yield via OXPHOS (**Figure 2E**). Furthermore, mitochondrial fragmentation has been shown to contribute to the production of inflammatory cytokines and ROS, both of which play critical roles in antimicrobial and inflammatory responses (57). These findings are consistent with our results, which demonstrate reduced ATP levels, elevated ROS, and increased production of TNF-α, NO and IL-6 (**Figure 2I**) when macrophages are exposed to EcN-OMVs. Lastly, we found that EcN-OMV exposure elevated iNOS expression and activated the NF-κB pathway both of which are related to inflammation. iNOS is essential for sustained high NO production. In macrophages, NO can function as a cytotoxic agent against infectious microorganisms (58) or serve as an immunoregulator (59). It has been reported that macrophages in the M1-polarized state express iNOS, with its expression usually regulated by the transcription factor NF-κB (59) which is essential for inflammatory responses.

Our KEGG enrichment analysis indicated activation of the NF-κB pathway (**Figure 5D**), which was further confirmed by a significant increase in p65 protein levels (**Figure 5F**), a key regulator of the NF-κB pathway. Interestingly, activation of the NF-κB signaling pathway has been reported to promote macrophage migration (60), consistent with our results with EcN-OMVs. In summary, metabolic reprogramming of macrophages following EcN-OMV treatment, whether through activation of HIF-1 and NF-κB pathways, upregulation of glycolysis, suppression of the TCA cycle, or accumulation of ROS and NO is essential for macrophage proliferation, inflammation, phagocytosis, and migration.

## 5 Limitations

Although previous studies have confirmed the regulatory role of metabolism in macrophage polarization, metabolomics provides new insights into how proinflammatory phenotypes develop and affect macrophage function. However, the complex nature of intracellular metabolic pathways means that changes in intermediate metabolites are interdependent, and the regulatory balance within these pathways remains unclear. Macrophages are also influenced by a dynamic microenvironment, where the availability of metabolites and regulation of key enzymes can vary during polarization, complicating the identification of specific pathway roles. Additionally, this study is limited by using RAW 264.7 cells, which, lack the physiological relevance of primary macrophages. Further research is needed to validate these findings in primary macrophage models and to explore the time-dependent effects of EcN-OMV-induced polarization.

## 6 Conclusion

Based on metabolomic, fluxomic, and transcriptomic analyses, our study demonstrates that OMVs from probiotic *E. coli* Nissle 1917 induce M1 macrophage polarization by upregulating glycolysis and downregulating the TCA cycle. Furthermore, our findings indicate that metabolites present in *E. coli* Nissle 1917 OMVs, such as succinate, stearic acid, and BCAAs, are associated with macrophage proliferation, phagocytosis, and migration. This investigation establishes a theoretical foundation for utilizing probiotic OMVs in immunomodulation for therapeutic applications.

## Data Availability

The RNA-seq data generated in this study have been deposited in the NCBl Sequence Read Archive (SRA) with the accession number PRJNA1157568. Metabolomics data supporting the findings of this research are available from the corresponding authors upon request.

## Supplementary material

Supplementary Table 1 The concentration of metabolites in EcN-OMVs.

Supplementary Table 2 Statistical comparison of extracellular, intracellular, and biomass metabolite profiles between OMV and CON groups (P-values and fold changes).

Supplementary Figure 1 Apoptosis in RAW264.7 macrophages after EcN-OMV treatment measured with a trypan blue exclusion assay (n = 6).

Supplementary Figure 2
^13^C-labelling of extracellular metabolites. Bar charts show the incorporation of ^13^Carbon derived from ^13^C-glucose into metabolites in RAW264.7 cells with (blue) or without (grey) EcN-OMV treatment. M denotes the main molecular ion of identified metabolites. M^+^1 is 1 m/z higher than the M. Statistical significance was determined using the student’s t-test: *p < 0.05; **p < 0.01; ***p < 0.001; ****p < 0.0001.

Supplementary Figure 3
^13^C-labelling of intracellular metabolites. Bar charts show the incorporation of ^13^Carbon derived from ^13^C-glucose into metabolites in RAW264.7 cells with (red) or without (grey) EcN-OMVs treatment. M denotes the main molecular ion of identified metabolites. M+1 is 1 m/z higher than the M. Statistical significance was determined using the students t-test: *p < 0.05; **p < 0.01; ***p < 0.001; ****p < 0.0001.

Supplementary Figure 4
^13^C-labelling of biomass metabolites. Bar charts show the incorporation of ^13^Carbon derived from ^13^C-glucose into metabolites in RAW264.7 cells with (red) or without (grey) EcN-OMVs treatment. M denotes the main molecular ion of identified metabolites. M+1 is 1 m/z higher than the M. Statistical significance was determined using the students t-test: *p < 0.05; **p < 0.01; ***p < 0.001; ****p < 0.0001.
