# Supplementary material for: Outer membrane vesicles derived from probiotic Escherichia coli Nissle 1917 promote metabolic remodeling and M1 polarization of RAW264.7 macrophages
Source: Front Immunol. 2025 May 29;16:1501174. doi: 10.3389/fimmu.2025.1501174 (PMC12159019; doi:10.3389/fimmu.2025.1501174)
Supplement: Supplementary file 1 [file DataSheet1.doc]

**Supplementary Material: R code**

**1.** **Lollipop chat**

setwd("Folder path")

dir()

library(ggplot2)

dfm <-read.csv("data.csv")

names(dfm)

# Convert the cyl variable to a factor

dfm$Classfication <- as.factor(dfm$Classfication)

library(ggpubr)

ggdotchart(dfm, x = "Name", y = "aver",

color = "Classfication",

palette=c("#2F7FC1","#EE7072", "#96C37D","#C497B2","#9DC3E7","#F0988C","#6B70B0"),

sorting = "asc", sort.by.groups = TRUE,

add = "segments",

add.params = list(color = "gray90", size = 0.8),

group = "Classfication",

dot.size = 2,

ggtheme = theme_pubclean()

) + font("x.text", size = 6) + facet_wrap(~Type,scales="free_y", ncol=1) +

theme(strip.background= element_rect(fill="grey95"))

**2.PCA analysis**

runPCA <-function(){

PCA<-function(conditions=1:3, ball.size=0.05, text.size = 1, log=TRUE){

require(rgl)

report_folder <<- tk_choose.dir(caption = "Select the working directory")

setwd(report_folder)

original.df<- tk_choose.files(caption = "Select metabolite or pathways profile in .csv")

final.df <- read.csv(original.df)

final.df[is.na(final.df)]<-0

final.stat <- final.df

abc <- 1:100

options("menu.graphics"=TRUE)

selected.samples <- numeric()

for (q in 1:length(conditions)) {

cat(paste("Select samples from condition:", q, "\n"))

samples <- select.list(names(final.stat), multiple = TRUE,

title = paste("Samples in condition", q))

columns <- names(final.stat)

equal <- 0

equal_list <- 0

for (t in 1:length(samples)) {

equal <- which(columns == samples[t])

if (length(equal) != 0) {

equal_list <- rbind(equal_list, equal)

}

}

equal_list <- (equal_list[-1])

names(final.stat)[equal_list] <- abc[q]

if (length(selected.samples) == 0) {

selected.samples <- equal_list

}

else {

selected.samples <- c(selected.samples, equal_list)

}

}

group <- names(final.stat)[selected.samples]

data.df<- final.stat[,selected.samples]

names(data.df)<-names(final.df[,selected.samples])

group<-as.numeric(group)

replicates <- group[!duplicated(group)]

colur.panel<-rainbow(length((replicates)))

colour<-NULL

for(i in 1:length(group)){

colour<- c(colour,colur.panel[group[i]])

}

colour_replot<<-colour

cat(paste("PCA analysis is in progress..", "\n"))

data.replot<<-data.df

if(log==TRUE){

data.df[is.na(data.df)]<-0

data.df<-log(data.df)

data.df[data.df=="-Inf"]<-0

}

pc1<-prcomp(data.df)

PC<-pc1$r

**3.heatmap**

library(RColorBrewer)

library(pheatmap)

library(ComplexHeatmap)

library(circlize)

setwd("Folder path ")

dir()

data.df <-read.csv("data.csv", header= TRUE)

data_1.df<-(data.df[,-c(1:2)])

heat <- (as.matrix(data_1.df))

heat[is.na(heat)] <- 0

rownames(heat) <-data.df[,1]

annotation_col<-data.frame(Groups= factor(rep(c("Alanine/Control","NA/Control","Pyr/Control","GLA/Control"),c(1,1,1,1)))) # change group names

annotation_row <- data.frame(MetaboliteClass=data.df[,2])

rownames(annotation_row)<-data.df[,1]

unique(data.df[,2]) # number of classification

Ann_colors = list(

Groups = brewer.pal(4, "Set2")

)

Ann_colors$MetaboliteClass <- brewer.pal(7, "Set3")

names(Ann_colors$Groups) <- unique(annotation_col$Groups)

names(Ann_colors$MetaboliteClass) <- unique(data.df[, 2])

length(unique(data.df[, 2]))

names(Ann_colors$MetaboliteClass) <- unique(data.df[, 2])

names(Ann_colors$Groups) <- unique(annotation_col$Groups)

names(Ann_colors$MetaboliteClass) <- unique(data.df[,2])

paletteLength <- 200

myColor <- colorRampPalette(c("lightskyblue3", "white", "indianred3"))(paletteLength) # change the colour your preference

myColor <-colors <- colorRampPalette(rev(brewer.pal(n = 5, name = "RdYlBu")))(paletteLength)

myBreaks <- c(seq(min(heat), 0, length.out=ceiling(paletteLength/2) + 1),

seq(max(heat)/paletteLength, max(heat), length.out=floor(paletteLength/2)))

pheatmap(heat,

annotation_col = annotation_col,

cluster_cols = FALSE,

annotation_row = annotation_row,

annotation_colors = Ann_colors,

row_split = annotation_row$MetaboliteClass,

column_split = annotation_col$Groups,

border_color = "white",

color = myColor,

breaks = myBreaks,

fontsize_row = 14,

fontsize_col = 12,

angle_col = "45"

)

5**.** **Volcano plot**

library(EnhancedVolcano)

setwd("Folder path ")

dir()

mydata <- read.csv("data.csv")

head(mydata, 10)

EnhancedVolcano(mydata,

x = "log2FoldChange",

y = "pvalue",

lab = mydata$Gene

)

colnames(mydata)

summary(mydata$name)

table(is.na(mydata$name))

mydata$name <- as.character(mydata$metabolites)

mydata$name[is.na(mydata$name)] <- "Unknown"

EnhancedVolcano(mydata,

x ="log2FoldChange",

y ="pvalue",

lab = mydata$metabolites,

pCutoff = 0.001, # p-value

FCcutoff = 1, # fold change

pointSize = 2.0,

labSize = 2.5,

colAlpha = 0.7,

gridlines.major = FALSE,

gridlines.minor = FALSE,

cutoffLineCol="red",

)

EnhancedVolcano(mydata,

x ="log2FoldChange",

y ="pvalue",

lab = mydata$metabolites,

pCutoff = 0.05,

FCcutoff = 1.5,

pointSize = 2.0,

labSize = 3,

colAlpha = 0.4,

col = c("#01bb17", 'black', 'skyblue', 'red3'),

gridlines.major = FALSE,

gridlines.minor = FALSE,

boxedLabels = TRUE,

labCol = 'black',

labFace = 'bold',

cutoffLineCol="grey"

colConnectors = 'black',

drawConnectors = TRUE,

widthConnectors = 0.75

)

**6. Metabolic pathway enrichment analysis**

setwd("Folder path")

dir()

data.df<- read.csv( "data.csv")

names(data.df)

metabolite.names <-as.character(data.df[,1])

Class <-as.character(data.df[,2])

sample_type <- as.character(data.df[,5])

pvalue<- log(data.df$pvalue,2)

NumberOfMetabolites <- data.df$Hit

Contr_Pvalue <- rep(10,length(pvalue))

Contr_NumberOfMetabolites <- rep(1,length(NumberOfMetabolites ))

data.df_1<-data.df[,c(3,4)]

names(data.df_1)

Treatments<-c("Control", "1_PECOS")

data.df_mean <- t(data.df_1)

my_frame<-NULL

for(i in seq(along=data.df_mean[1,])) {

my_frame<-rbind(my_frame, data.frame(metabolite_names=rep(metabolite.names[i],nrow(data.df_mean)),

Classification = rep(Class[i],nrow(data.df_mean)),

sampletype=rep(sample_type[i],nrow(data.df_mean)),

Aboundance=data.df_mean[,i],

Treatments=Treatments,

pvalue=c(Contr_Pvalue[i],pvalue[i]), Metabolites_Number=c(Contr_NumberOfMetabolites[i],NumberOfMetabolites[i])))

}

rownames(my_frame)<-NULL

my_frame

library(ggplot2)

theme_set(theme_bw())

library(Hmisc)

my_frame[is.na(my_frame)]<-0

my_frame2<- within(my_frame, {

metabolite_names <-reorder(metabolite_names, Aboundance)

Classification <- reorder(Classification, -Aboundance)

})

library(scales)

myplot<-ggplot(my_frame2, aes(x=Aboundance, y=metabolite_names, colour = pvalue,

group=Treatments), na.rm=TRUE)

A <- geom_point(alpha=I(0.9),(aes(size = Metabolites_Number)))

B <- scale_colour_brewer(palette="Set1")

C <- facet_grid(Classification~sampletype,scales="free_y",space = "free")

D <- theme(strip.text.y = element_text(size=9))

E <- theme(strip.background= element_rect(fill="grey98",colour="grey70"))

F <- labs(x="Activity: (-) Down-regulation, (+) Up-regulation",y="Metabolite pathway")

H <- geom_line(alpha=I(0.3))

I <- theme(axis.text.y= element_text(size =9))

J <- theme(axis.text.x= element_text(size =9))

k <- theme(strip.text.y=element_text(angle=360,size=9))

n <- theme(panel.grid.minor = element_blank(),panel.grid.major = element_blank())

p <- scale_colour_gradient2(low = "red", mid='white', high = "black", midpoint = 0)

myplot + H+ A + B + C + D + E + F + I + J + k + n + p

setwd("Folder path")

dir()

library(GOplot)

data.df<-read.csv("data.csv", row.names="Name")

GOChord(data.df,nlfc =0, space = 0.02, gene.space =0.5 ,gene.size =5,process.label=5 )
