# Supplementary material for: Outer membrane vesicles derived from probiotic Escherichia coli Nissle 1917 promote metabolic remodeling and M1 polarization of RAW264.7 macrophages
Source: Front Immunol. 2025 May 29;16:1501174. doi: 10.3389/fimmu.2025.1501174 (PMC12159019; doi:10.3389/fimmu.2025.1501174)
Supplement: Supplementary Table 1 — The concentration of metabolites in EcN-OMVs. [file Table1.doc]

**Supplementary materials**

**Supplementary Table 1. The concentration of metabolites in EcN-OMVs**

| **Metabolite** |  | Concentration(mM)/Exosome x10^9^ |
| --- | --- | --- |
| **Amino Acids** |  |  |
| Aspartic acid |  | 3625.52 |
| Oxidized Glutathione |  | 1277.77 |
| Glutamic acid |  | 763.13 |
| Lysine |  | 501.48 |
| Isoleucine |  | 415.20 |
| Valine |  | 335.07 |
| Alanine |  | 332.19 |
| Proline |  | 309.36 |
| Tyrosine |  | 308.32 |
| Glycine |  | 286.21 |
| Methionine |  | 242.96 |
| N-Acetyl-L-aspartic acid |  | 101.40 |
| Serine |  | 7.62 |
| Threonine |  | 5.93 |
| N-Acetly-L-phenylalanine |  | 1.83 |
| **Fatty Acids** |  |  |
| Stearic acid |  | 1956.49 |
| Heneicosanoic acid |  | 86.43 |
| Dodecanoic acid |  | 81.76 |
| Hexanoic acid |  | 69.10 |
| Tridecanoic acid |  | 53.87 |
| Margaric acid |  | 20.42 |
| Octanoic acid |  | 14.04 |
| Arachidic acid |  | 13.29 |
| Behenic acid |  | 5.60 |
| Lignoceric acid |  | 1.39 |
| Undecanoic acid |  | 0.86 |
| Tricosanoic acid |  | 0.42 |
| Stearic acid |  | 1956.48 |
| Heneicosanoic acid |  | 86.43 |
| Dodecanoic acid |  | 81.76 |
| Hexanoic acid |  | 69.10 |
| Erucic acid |  | 196.02 |
| DHA |  | 57.78 |
| 10-Heptadecenoic acid |  | 51.08 |
| Palmitelaidic acid |  | 25.47 |
| Linoleic acid |  | 13.89 |
| trans-Vaccenic acid |  | 8.27 |
| Arachidonic acid |  | 5.62 |
| **TCA Intermediates** |  |  |
| Succinic acid |  | 238.44 |
| Lactic acid |  | 129.95 |
| cis-Aconitic acid |  | 17.09 |
| Pyruvic acid |  | 4.76 |
| a-Ketoglutaric aicd |  | 2.17 |
| Malic acid |  | 1.89 |
| DL-Isocitric acid |  | 1.51 |
| Citric acid |  | 1.34 |
| Fumaric acid |  | 0.74 |
| Itaconic acid |  | 0.18 |
| **Tryptophan Derivatives** |  |  |
| Indole |  | 23.86 |
