# Supplementary material for: Outer membrane vesicles derived from probiotic Escherichia coli Nissle 1917 promote metabolic remodeling and M1 polarization of RAW264.7 macrophages
Source: Front Immunol. 2025 May 29;16:1501174. doi: 10.3389/fimmu.2025.1501174 (PMC12159019; doi:10.3389/fimmu.2025.1501174)
Supplement: Supplementary Table 2 — Statistical comparison of extracellular, intracellular, and biomass metabolite profiles between OMV and CON groups (P-values and fold changes). [file Table2.doc]

**Supplementary Table 2. Statistical comparison of extracellular, intracellular, and biomass metabolite profiles between OMV and CON groups (P-values and fold changes)**

|  | **Extracellular** | | **Intracellular** | | **Biomass** | |
| --- | --- | --- | --- | --- | --- | --- |
|  | **OMV/CON** | | **OMV/CON** | | **OMV/CON** | |
| **Metabolite** | ***P* value** | **FC** | ***P* value** | **FC** | ***P* value** | **FC** |
| **Amino Acids** |  |  |  |  |  |  |
| 2-Hydroxybutyric acid | 0.0005 | 0.2830 | 0.9940 | 0.0000 | 0.0002 | -0.9642 |
| 3-Methyl-2-oxopentanoic acid | 0.4361 | 0.4067 | 0.1606 | 0.0000 | 0.0003 | -0.9374 |
| Alanine | 0.0258 | 0.3674 | 0.0019 | 0.2774 | 0.0218 | -0.6928 |
| Creatinine | 0.9510 | 0.0000 | 0.5489 | 0.0000 | 0.0197 | -0.0787 |
| Glutamic acid | 0.0000 | 0.7848 | 0.0046 | 0.0000 | 0.4605 | 0.0000 |
| Histidine | 0.0015 | 0.7777 | 0.8931 | 0.0000 | 0.5388 | 0.0000 |
| Homocysteine |  | 0.0000 | 0.0000 | -1.3406 | 0.0046 | -1.6758 |
| Isoleucine | 0.2518 | 0.0000 | 0.0006 | 0.2434 | 0.8630 | 0.0000 |
| L-alpha-Aminobutyric acid | 0.0003 | 0.3488 | 0.0022 | -0.3396 | 0.0006 | -1.5680 |
| Leucine | 0.0754 | 0.0000 | 0.0002 | 0.2992 | 0.0017 | -0.8568 |
| L-Hydroxyproline | 0.0406 | 0.3836 | 0.0001 | -0.7698 | 0.0147 | -1.2816 |
| Lysine | 0.1295 | 0.0000 | 0.1445 | 0.0000 | 0.0187 | -0.9398 |
| Methionine | 0.1513 | 0.0000 | 0.0012 | 0.1783 | 0.0026 | -0.9413 |
| N-acetyl-L-aspartic acid | 0.0000 | 0.9072 | 0.0000 | -0.9193 | 0.0107 | -0.7714 |
| Norleucine | 0.0037 | 0.7243 | 0.7611 | 0.0000 | 0.0137 | -1.4096 |
| Ornithine | 0.0001 | 0.2888 | 0.0015 | -1.5234 | 0.0248 | -0.7510 |
| Oxidized Glutathione | 0.0121 | 0.7075 | 0.1455 | 0.0000 | 0.0133 | -1.2866 |
| Phenylalanine | 0.0547 | 0.0000 | 0.0082 | 0.1002 | 0.0017 | -0.9465 |
| Proline | 0.0027 | 0.5383 | 0.0308 | 0.0941 | 0.0043 | -0.7946 |
| Pyroglutamic acid | 0.5979 | 0.0000 | 0.8800 | 0.0000 | 0.0033 | -1.1828 |
| Sarcosine | 0.0053 | 0.2351 | 0.0000 | 0.1524 | 0.0164 | -0.3363 |
| Serine | 0.0000 | 1.2622 | 0.0000 | 0.9044 | 0.0636 | 0.0000 |
| S-methyl-lcysteine | 0.0001 | 1.0954 | 0.0032 | 0.3928 | 0.3384 | 0.0000 |
| Threonine | 0.3196 | 0.0000 | 0.2391 | 0.0000 | 0.0065 | -1.0782 |
| Tyrosine | 0.0812 | 0.0000 | 0.0866 | 0.0000 | 0.0034 | -0.9985 |
| Valine | 0.6468 | 0.0000 | 0.0031 | 0.1774 | 0.0037 | -0.9792 |
| **Alkane** |  |  |  |  |  |  |
| Decane |  | 0.0000 | 0.0011 | 0.2471 | 0.0032 | -0.0500 |
| Docosane |  | 0.0000 | 0.0036 | 0.1726 | 0.1947 | 0.0000 |
| Dodecane |  | 0.0000 | 0.0019 | 0.2214 | 0.0014 | 0.1082 |
| Heptadecane |  | 0.0000 | 0.0000 | 0.6416 | 0.0054 | -0.0820 |
| Hexadecane |  | 0.0000 | 0.0010 | 0.2419 | 0.0035 | 0.0510 |
| Octadecane |  | 0.0000 | 0.0000 | 0.5222 | 0.0070 | -0.0382 |
| Tetradecane |  | 0.0000 | 0.0101 | 0.2398 | 0.0017 | 0.0649 |
| Tridecane |  | 0.0000 | 0.8170 | 0.0000 | 0.0268 | 0.0657 |
| Undecane |  | 0.0000 | 0.8240 | 0.0000 | 0.0013 | 0.1277 |
| **Fatty Acids** |  |  |  |  |  |  |
| 10-Heptadecenoic acid | 0.0234 | 0.3104 | 0.0532 | 0.0000 | 0.0056 | -0.1681 |
| 10-Pentadecenoic acid | 0.0006 | 0.1696 | 0.8931 | 0.0000 | 0.1339 | 0.0000 |
| 11,14,17-Eicosatrienoic acid | 0.0248 | 0.1817 | 0.0014 | 0.1818 | 0.0136 | -0.2672 |
| 11,14-Eicosadienoic acid | 0.0023 | 0.2904 | 0.0387 | 0.1774 | 0.4634 | 0.0000 |
| 11-Eicosenoic acid | 0.0224 | 0.2831 | 0.1947 | 0.0000 | 0.5333 | 0.0000 |
| 13,16-Docosadienoic acid | 0.5111 | 0.0000 | 0.0179 | 0.1680 | 0.3917 | 0.0000 |
| Adrenic acid | 0.0768 | 0.0000 | 0.0000 | 0.6410 | 0.0000 | 0.2205 |
| Arachidonic acid | 0.0259 | 0.2863 | 0.0400 | 0.1623 | 0.0010 | -0.0356 |
| Decanoic acid | 0.9402 | 0.0000 | 0.0263 | 0.1300 | 0.0003 | 0.0385 |
| DHA | 0.0410 | 0.2699 | 0.0406 | 0.2002 | 0.0007 | -0.0225 |
| Dodecanoic acid | 0.3328 | 0.0000 | 0.0000 | 0.2764 | 0.0001 | 0.1586 |
| DPA | 0.0257 | 0.2200 | 0.0054 | 0.2752 | 0.0011 | -0.0734 |
| Erucic acid | 0.3515 | 0.0000 | 0.0434 | 0.2209 | 0.0224 | -0.2334 |
| gamma-Linolenic acid | 0.2723 | 0.0000 | 0.1305 | 0.0000 | 0.0001 | 0.1104 |
| Heneicosanoic acid | 0.0999 | 0.0000 | 0.6425 | 0.0000 | 0.0473 | -0.3518 |
| Hexanoic acid | 0.0001 | 0.0557 | 0.2613 | 0.0000 | 0.0610 | 0.0000 |
| Homo-gamma-Linolenic acid | 0.4267 | 0.0000 | 0.0041 | 0.3502 | 0.0004 | -0.0120 |
| Lignoceric acid | 0.1813 | 0.0000 | 0.5509 | 0.0000 | 0.0322 | -0.7810 |
| Linoleic acid | 0.0306 | 0.3159 | 0.0468 | 0.1578 | 0.0000 | 0.2450 |
| Margaric acid | 0.0010 | 0.2344 | 0.0009 | 0.4320 | 0.0003 | 0.0136 |
| Myristic acid | 0.0001 | 0.0000 | 0.0005 | 0.0000 | 0.0336 | -0.6933 |
| Myristoleic acid | 0.0085 | 0.6009 | 0.0008 | -0.6165 | 0.0355 | -0.7185 |
| Nervonic acid |  | 0.0000 | 0.0084 | 0.2296 | 0.7382 | 0.0000 |
| Octanoic acid | 0.0494 | 0.3094 | 0.4487 | 0.0000 | 0.0017 | -0.0005 |
| Oleic acid | 0.0055 | 0.3212 | 0.1173 | 0.0000 | 0.0023 | -0.0005 |
| Palmitelaidic acid | 0.0000 | 1.0575 | 0.0409 | -0.3025 | 0.1545 | 0.0000 |
| Pentadecanoic acid | 0.0348 | 0.3132 | 0.1552 | 0.0000 | 0.0065 | -0.1811 |
| Stearic acid | 0.6058 | 0.0000 | 0.0047 | 0.2898 | 0.0015 | 0.0459 |
| trans-Vaccenic acid | 0.0406 | 0.3354 | 0.0040 | 0.2921 | 0.0016 | 0.0131 |
| Tricosanoic acid | 0.0008 | 0.8530 | 0.0412 | 0.0196 | 0.0060 | -0.1905 |
| Tridecanoic acid | 0.5880 | 0.0000 | 0.0155 | 0.0626 | 0.0040 | -0.1525 |
| Undecanoic acid | 0.2318 | 0.0000 | 0.0060 | 0.1454 | 0.0028 | -0.0249 |
| **Other** |  |  |  |  |  |  |
| 1,4-Benzenedicarboxylic acid | 0.0484 | 0.2561 | 0.0201 | 0.3642 | 0.4375 | 0.0000 |
| 11-Octadecenoic acid |  | 0.0000 | 0.4342 | 0.0000 | 0.0030 | -0.0004 |
| 1-Dodecanol |  | 0.0000 | 0.0020 | 0.3144 | 0.0059 | -0.0867 |
| 2,4-Di-tert-butylphenol | 0.0085 | 0.2953 | 0.2632 | 0.0000 | 0.0370 | -0.1697 |
| Ala-Ala |  | 0.0000 | 0.0214 | -0.4435 | 0.0001 | -1.1249 |
| Benzeneacetic acid | 0.0008 | 0.3107 | 0.3166 | 0.0000 | 0.0008 | -0.9236 |
| Butanedioic acid | 0.1902 | 0.0000 | 0.0000 | -1.3231 | 0.0030 | -1.3708 |
| Cholesta-3,5-diene |  | 0.0000 | 0.0038 | 0.7285 | 0.0000 | 0.9587 |
| Cholesta-4,6-dien-3-ol |  | 0.0000 | 0.0024 | 0.8691 | 0.0001 | 0.6347 |
| Cholesterol |  | 0.0000 | 0.0405 | 0.2431 | 0.0061 | -0.0728 |
| cis-5,8,11-Eicosatrienoic acid |  | 0.0000 | 0.0020 | -0.4012 | 0.0886 | 0.0000 |
| dl-Chimyl alcohol |  | 0.0000 | 0.0214 | 0.1559 | 0.3964 | 0.0000 |
| Glycyl-l-proline |  | 0.0000 | 0.8769 | 0.0000 | 0.0006 | -1.1505 |
| Hexachlorethane | 0.0049 | -0.0613 | 0.0013 | 0.2207 | 0.0018 | 0.0436 |
| Hexadec-9-enoate |  | 0.0000 | 0.9440 | 0.0000 | 0.0021 | 0.1515 |
| Malonic acid |  | 0.0000 | 0.0405 | -0.3165 | 0.0004 | -0.8523 |
| N-Acetyl-phenylalanine |  | 0.0000 | 0.1875 | 0.0000 | 0.0013 | -1.3956 |
| n-Hexadecanoic acid | 0.0021 | 0.2940 | 0.4199 | 0.0000 | 0.0020 | 0.1556 |
| a-Ketoglutaric aicd | 0.2359 | 0.0000 | 0.0002 | -2.0152 | 0.0000 | -1.3969 |
| cis-Aconitic acid | 0.0000 | -0.3716 | 0.3625 | 0.0000 | 0.1904 | 0.0000 |
| Citric acid | 0.0000 | -0.4148 | 0.5798 | 0.0000 | 0.4301 | 0.0000 |
| DL-Isocitric acid | 0.0001 | 0.7194 | 0.0022 | -0.6629 | 0.1281 | 0.0000 |
| Fumaric acid | 0.5796 | 0.0000 | 0.0003 | -0.9709 | 0.0321 | -1.0606 |
| Itaconic acid | 0.0000 | 1.2116 | 0.0712 | 0.0000 | 0.1749 | 0.0000 |
| Lactic acid | 0.0284 | 0.2298 | 0.0081 | 0.2190 | 0.6521 | 0.0000 |
| **TCA Intermediates** |  |  |  |  |  |  |
| Malic acid | 0.2102 | 0.0000 | 0.0001 | -0.7767 | 0.0021 | -0.8326 |
| Pyruvic acid | 0.0275 | -0.2199 | 0.0012 | -1.3168 | 0.0179 | -1.1759 |
| Succinic acid | 0.0322 | 0.3077 | 0.0000 | -0.8099 | 0.0010 | -1.0954 |
| **Tryptophan Derivatives** |  |  |  |  |  |  |
| gamma-Aminobutyric acid |  | 0.0000 | 0.0005 | -0.5975 | 0.0386 | -0.9473 |
| Kynurenine |  | 0.0000 | 0.0040 | 0.1171 | 0.0351 | -0.2769 |
| Picolinic acid |  | 0.0000 | 0.0085 | 0.2154 | 0.0086 | -0.0765 |
