# Supplementary figures and images for: Outer membrane vesicles derived from probiotic Escherichia coli Nissle 1917 promote metabolic remodeling and M1 polarization of RAW264.7 macrophages
Source: Front Immunol. 2025 May 29;16:1501174. doi: 10.3389/fimmu.2025.1501174 (PMC12159019; doi:10.3389/fimmu.2025.1501174)

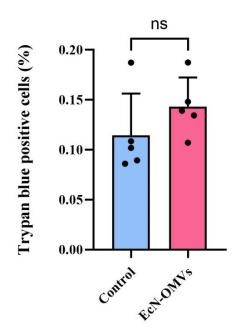

Supplement: Supplementary Figure 1 — Apoptosis in RAW264.7 macrophages after EcN-OMV treatment measured with a trypan blue exclusion assay (n = 6). [file Image1.jpeg]

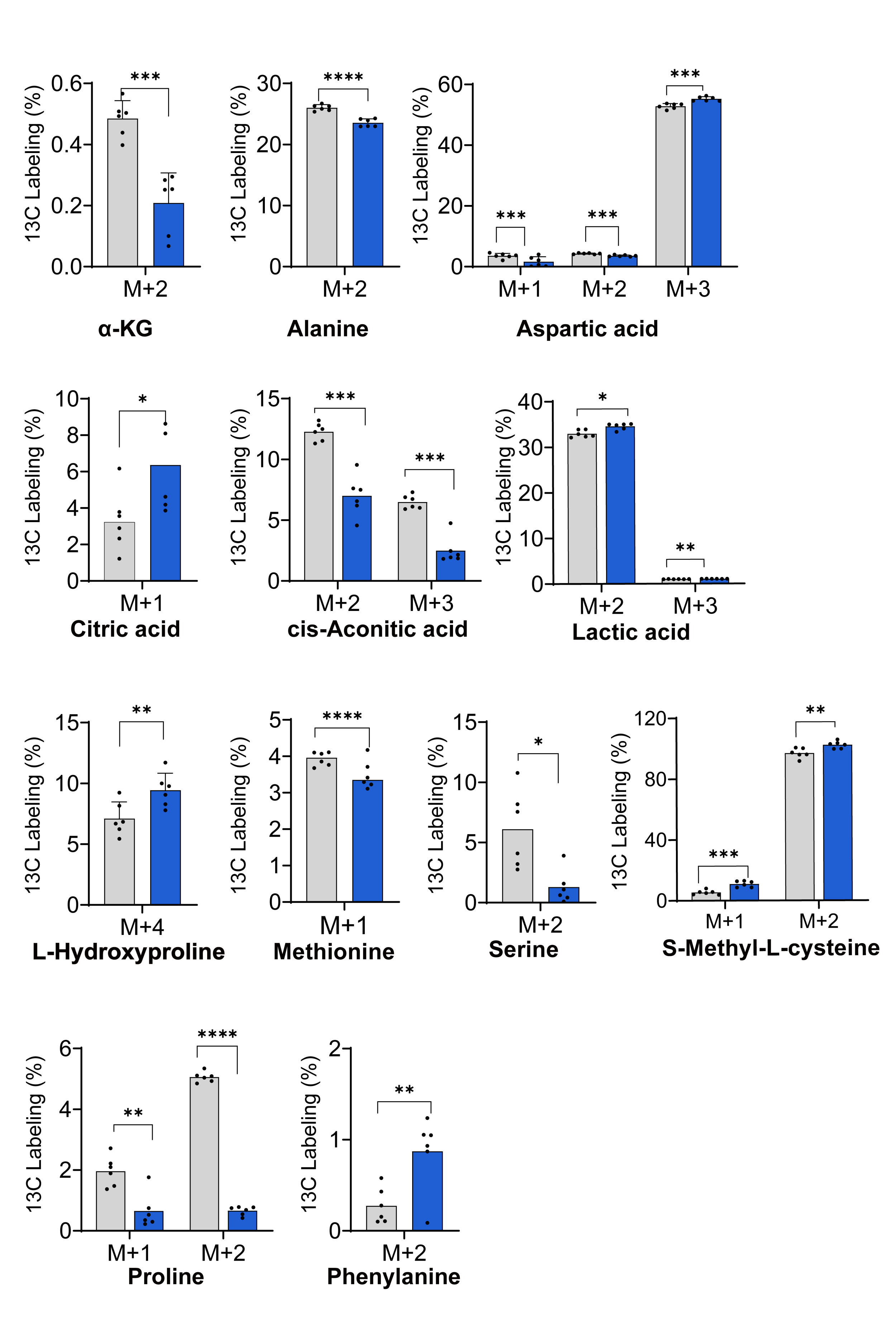

Supplement: Supplementary Figure 2 — 13C-labelling of extracellular metabolites. Bar charts show the incorporation of 13Carbon derived from 13C-glucose into metabolites in RAW264.7 cells with (blue) or without (grey) EcN-OMV treatment. M denotes the main molecular ion of identified metabolites. M+1 is 1 m/z higher than the M. Statistical significance was determined using the student’s t-test: *p < 0.05; **p < 0.01; ***p < 0.001; ****p < 0.0001. [file Image2.jpeg]

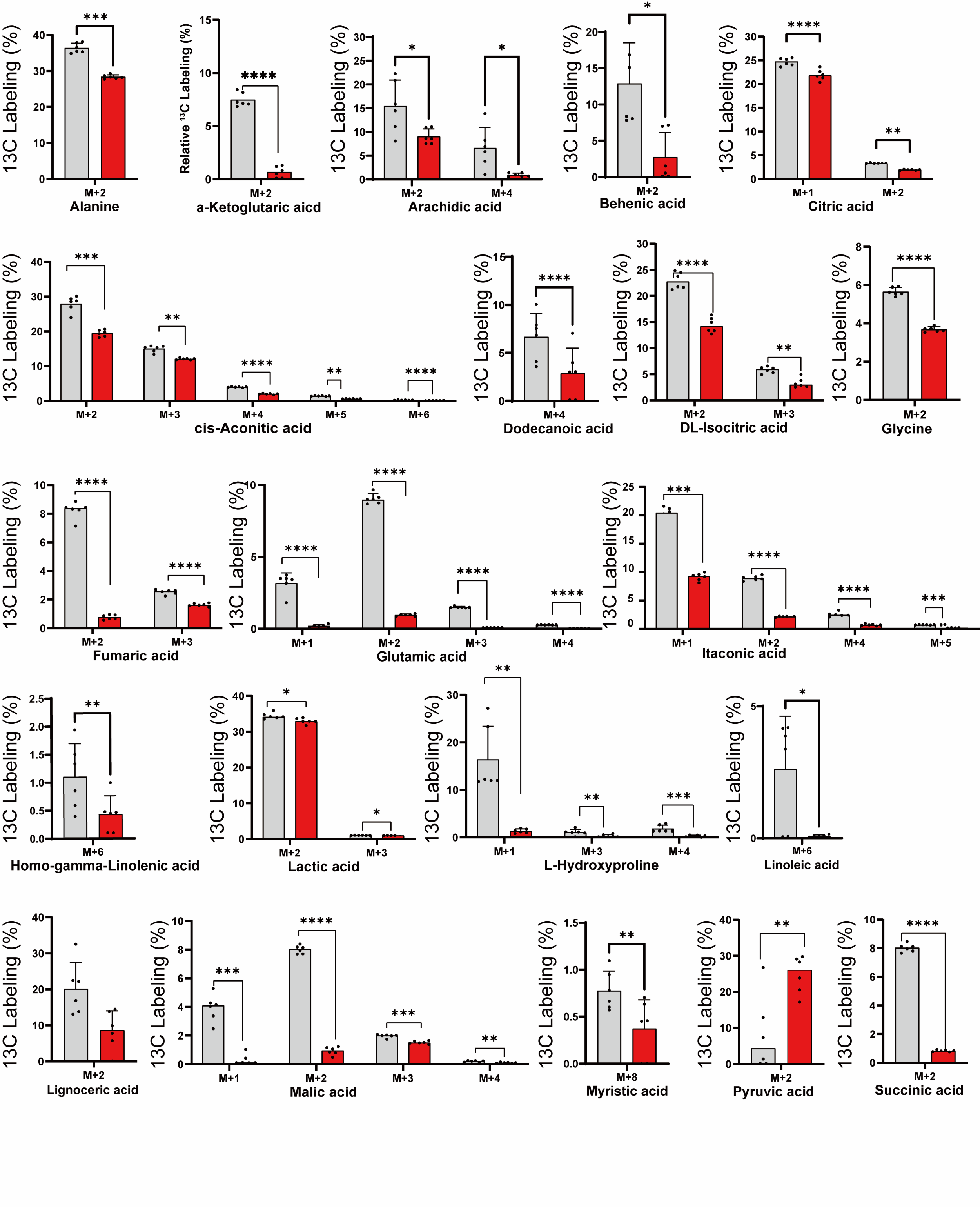

Supplement: Supplementary Figure 3 — 13C-labelling of intracellular metabolites. Bar charts show the incorporation of 13Carbon derived from 13C-glucose into metabolites in RAW264.7 cells with (red) or without (grey) EcN-OMVs treatment. M denotes the main molecular ion of identified metabolites. M+1 is 1 m/z higher than the M. Statistical significance was determined using the students t-test: *p < 0.05; **p < 0.01; ***p < 0.001; ****p < 0.0001. [file Image3.jpeg]

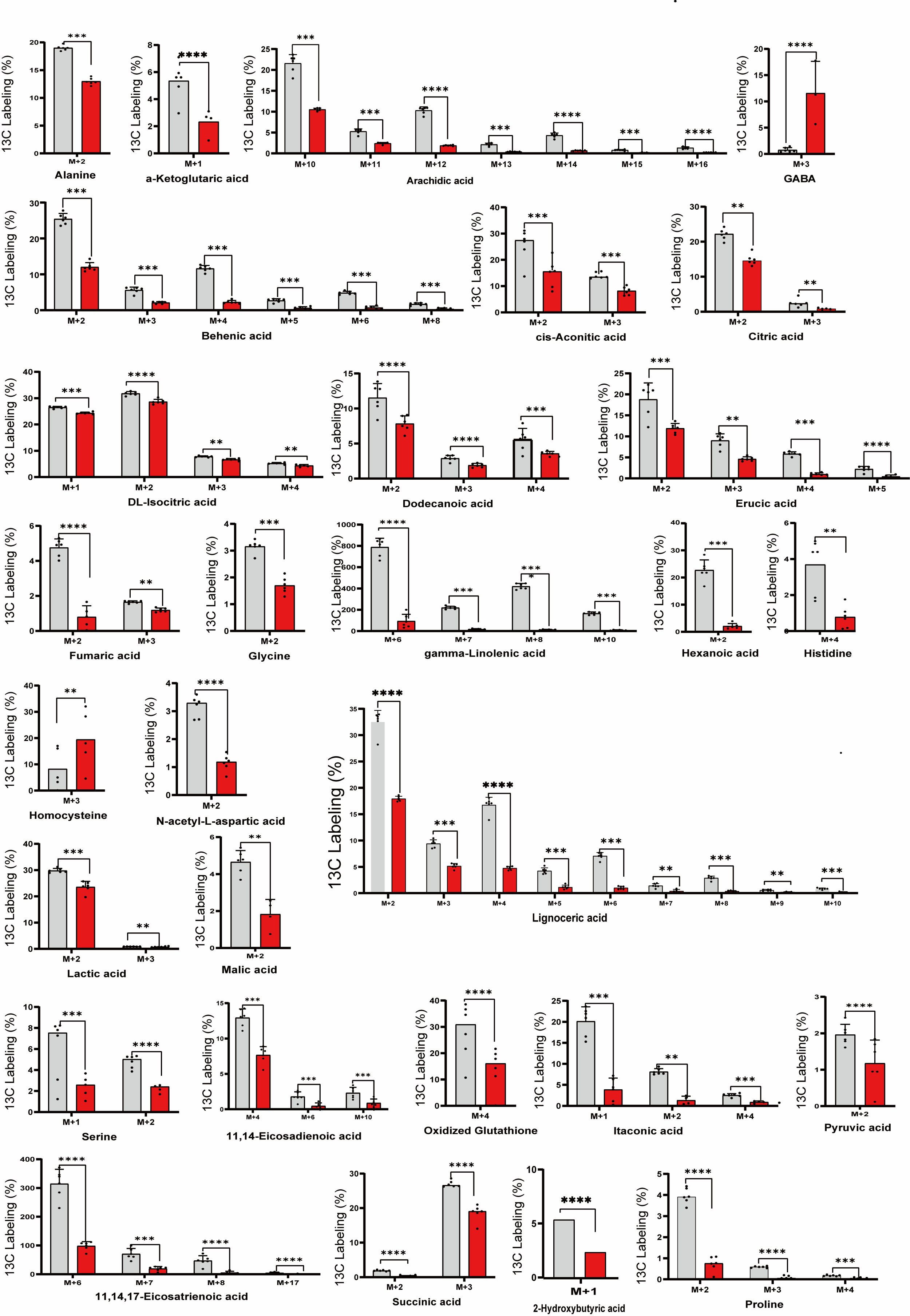

Supplement: Supplementary Figure 4 — 13C-labelling of biomass metabolites. Bar charts show the incorporation of 13Carbon derived from 13C-glucose into metabolites in RAW264.7 cells with (red) or without (grey) EcN-OMVs treatment. M denotes the main molecular ion of identified metabolites. M+1 is 1 m/z higher than the M. Statistical significance was determined using the students t-test: *p < 0.05; **p < 0.01; ***p < 0.001; ****p < 0.0001. [file Image4.jpeg]

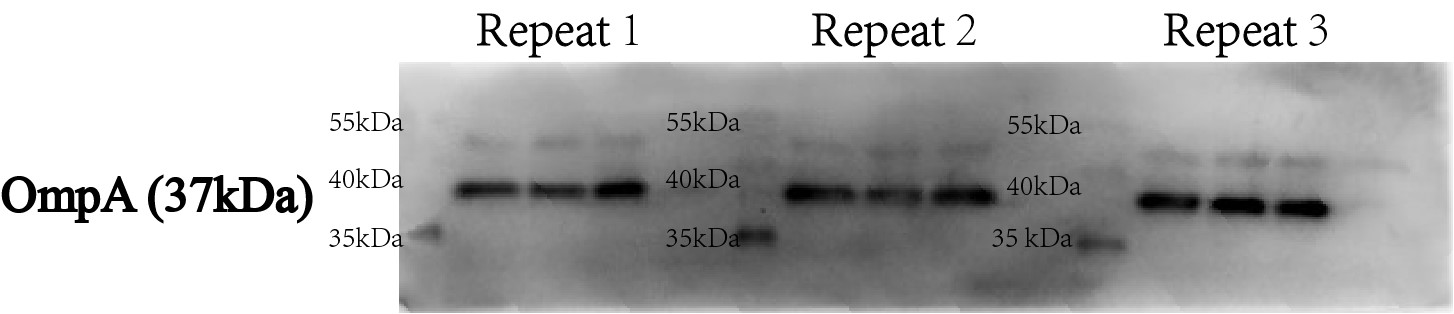

Supplement: Supplementary file 8 [file Image5.jpeg]

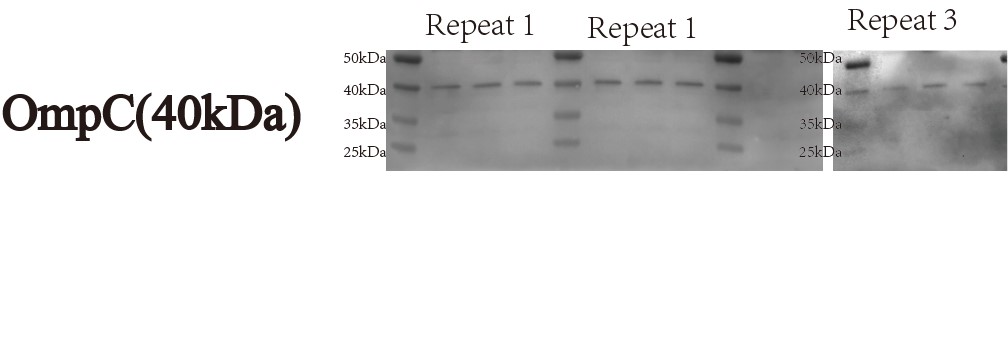

Supplement: Supplementary file 9 [file Image6.jpeg]

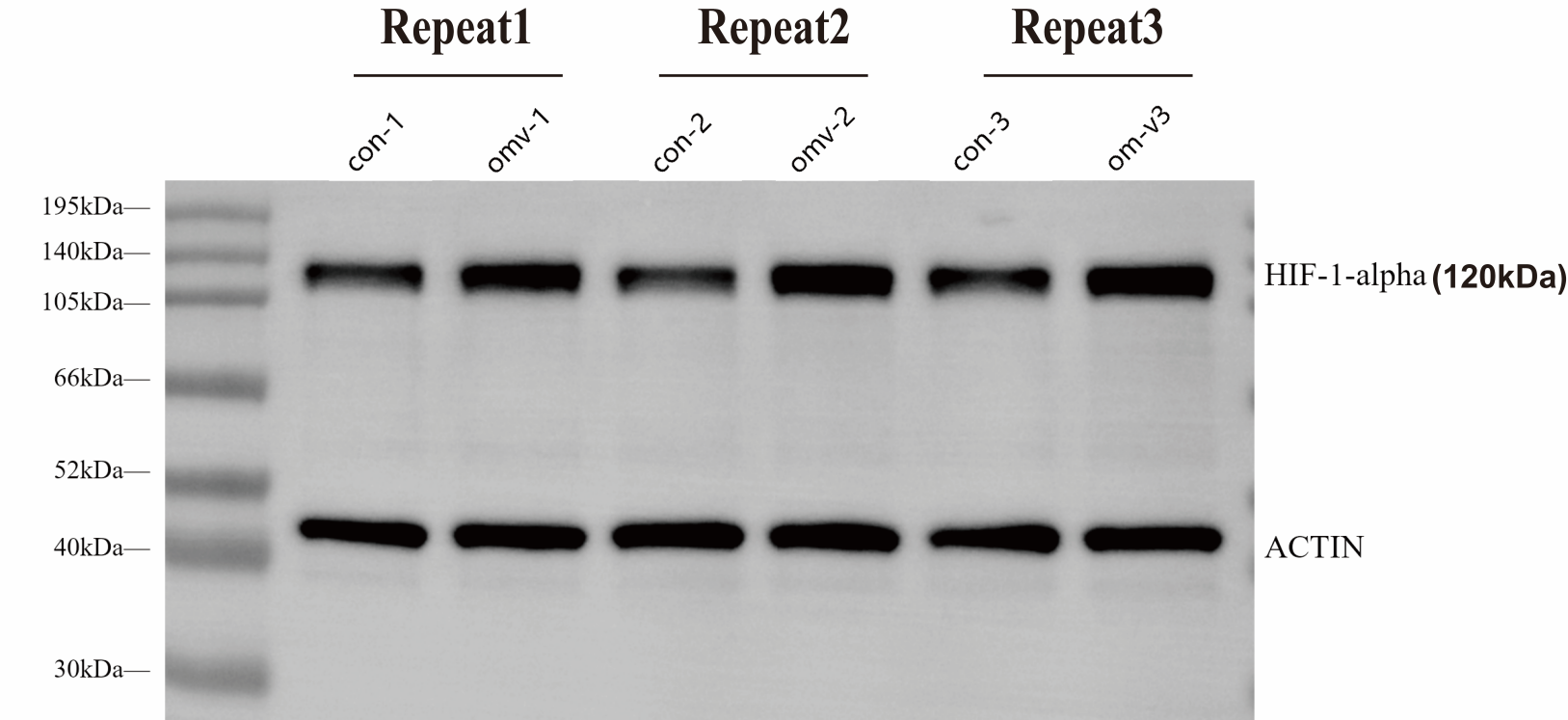

Supplement: Supplementary file 10 [file Image7.png]

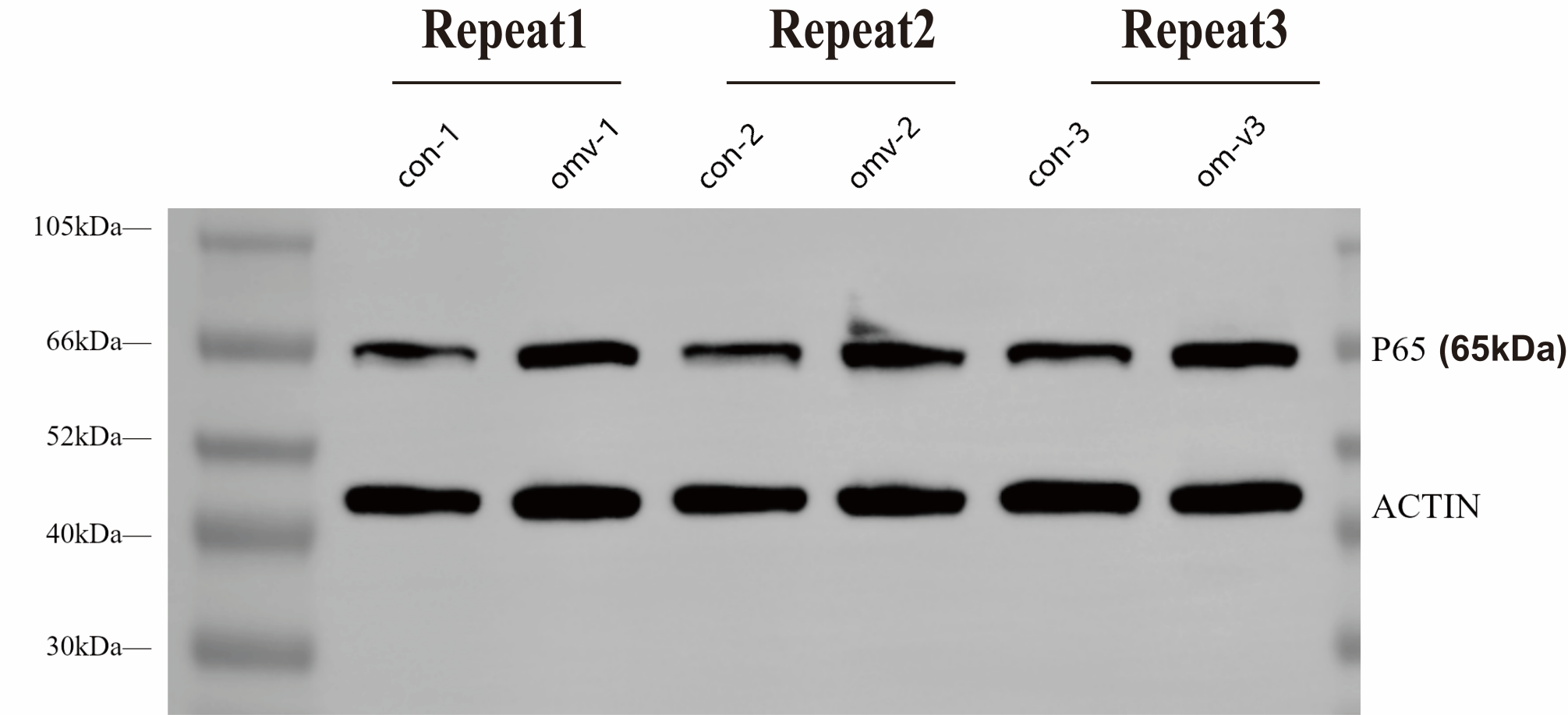

Supplement: Supplementary file 11 [file Image8.png]
